# Versatile Co_3_O_4_@metal oxide nanocomposites: from photocatalytic pollutant degradation to high-efficiency energy supercapacitor storage

**DOI:** 10.1039/d6ra06628c

**Published:** 2026-09-10

**Authors:** Ahmed Souemti, Radhouene Kahlaoui, Abderraouf Jraba, Muhammad Humayun, Mohamed Bououdina, Adel Megriche, Latifa Latrous

**Affiliations:** a Laboratory of Applied Inorganic Chemistry, LR19ES02, Department of Chemistry, Faculty of Sciences of Tunis, Tunis El Manar University El Manar 1 Tunis 2092 Tunisia ahmed.souemti@fst.utm.tn; b Preparatory Institute for Engineering Studies of Gafsa, Gafsa University El Khayzorane-Zarroug Street Gafsa 2112 Tunisia; c University of Carthage, Faculty of Sciences of Bizerte, Chemistry Department 7021 Zarzouna Bizerte Tunisia; d Laboratory of the Application of Materials to Environment, Water, and Energy (LAM3E) LR21ES15, Gafsa University, Faculty of Sciences of Gafsa, Department of Chemistry Sidi Ahmed Zarroug 2112 Gafsa Tunisia; e Energy, Water and Environment Lab, College of Sciences and Humanities, Prince Sultan University Riyadh 11586 Saudi Arabia; f Preparatory Institute for Engineering Studies of El Manar, Tunis El Manar University El Manar 1, B.P. 244 Tunis 2092 Tunisia

## Abstract

Co_3_O_4_-based heterojunction nanocomposites (Co_3_O_4_@M-O_*x*_; where M = Zn, Ti or Cu) were synthesized using a simple, cost-effective, eco-friendly hydrothermal method. These nanocomposites can be used in a variety of applications. Characterisation using X-ray diffraction (XRD), Fourier-transform infrared spectroscopy (FTIR), X-ray photoelectron spectroscopy (XPS), diffuse reflectance spectroscopy, high-resolution transmission electron microscopy (HRTEM), Brunauer–Emmett–Teller (BET) analysis and complex impedance spectroscopy (EIS) confirmed the presence of pure nanoscale phases measuring 76–91 nm and mesoporous type IV structures. The formation of successful p–n or p–p heterojunctions with spherical morphology was also confirmed. Band gap tuning revealed that Co_3_O_4_@CuO narrows the gap for visible-light photocatalysis, whereas Co_3_O_4_@ZnO and Co_3_O_4_@TiO_2_ widen it for high-energy redox applications. The composites achieved 90% degradation of Rhodamine B (RhB) and Methylene blue (MB) within 135 minutes (pseudo-first-order kinetics), which can be attributed to Fermi-level-driven band bending and the formation of internal electric fields that separate electrons and holes, thereby suppressing recombination and enhancing the generation of reactive oxygen species. Complex impedance spectroscopy (EIS) was used to characterise the electrical properties. Electrical conduction is thermally activated (100–200 °C) with higher activation energies (0.178–0.245 eV) reflecting the depletion-layer barriers at the grain boundaries. Thanks to interfacial synergy, these materials demonstrate improved conductivity and fast ion/electron transport, showing great promise for use in supercapacitors, water-splitting electrodes, zinc–air batteries, flexible solid-state storage and photocatalytic pollutant degradation.

## Abstract 2

Co_3_O_4_-M-O_*x*_ (M = Zn, Ti, and Cu) nanocomposites exhibit high efficiency in degrading organic dyes and shows semiconducting behavior at lower temperatures, making them suitable for capacitor applications in the field of energy storage.

## Graphical abstract

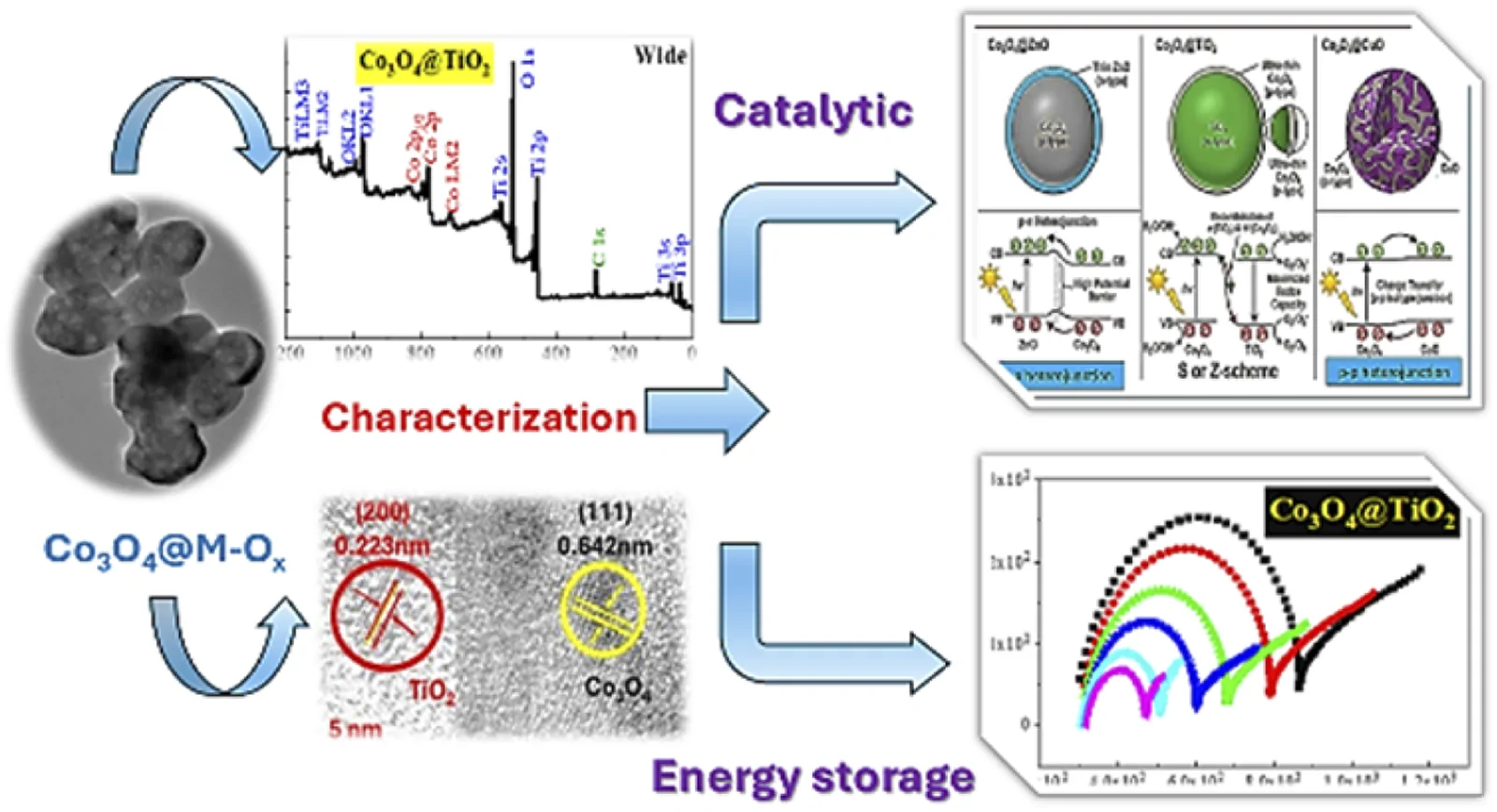

## Introduction

The development of materials for various applications that are both economically viable and inexpensive to synthesise is continuing to grow rapidly. Among these materials, heterojunction materials and heterostructure nanocomposites are important for next-generation technologies in the energy, environmental, and information technology fields. These nanocomposites overcome major trade-offs associated with single-phase materials by designing interfaces and hierarchical architectures, a principle demonstrated across polymer nanocomposites (You *et al.*), magnetic adsorbents (Melegy *et al.*), structural hybrids (Thiru *et al.*), electromagnetic materials (Fahidazar *et al.*), and high-voltage applications (Haghir Madadi *et al.*).^1–5^

Recent advances in heterojunction photocatalysts have been reported across various material systems, including metal–organic frameworks (Dhakshinamoorthy and García), organic semiconductor nanoparticles (Kosco *et al.*) and two-dimensional (2D) transition metal dichalcogenides (Joseph *et al.*). These heterojunctions improve charge separation and limit recombination, significantly increasing photocatalytic hydrogen production, carbon dioxide (CO_2_) reduction, dye degradation and solar fuel production.^6–8^

The study by Y. Zhu confirms that van der Waals heterostructures, which stack two-dimensional (2D) materials with zero-, one- and three-dimensional (0D, 1D and 3D) materials, can be used to create highly integrated photoelectric, memory and neuromorphic devices. These devices have tuneable electronic, optical and thermal properties and can be used for energy harvesting and brain-inspired computing.^9^ Another type of heterostructure, consisting of two-dimensional (2D) transition metal dichalcogenides synthesised by D. Nepal *et al.*, has a wide range of applications in optoelectronics, sensors, and photovoltaics. This overcomes the limitations of individual monolayers, paving the way for the development of advanced biosensing platforms.^10^

Transition metal oxides (TMOs), such as Co_3_O_4_, NiO, ZnO and MnO_2_, as well as perovskite and spinel structures, are versatile electrode materials and electrocatalysts due to their high electrochemical activity, low cost and adjustable composition. Their use in energy storage has been widely reviewed (Yuan *et al.*), their perovskite and spinel phases have been investigated in the context of electrochemical energy production (Flores-Lasluisa *et al.*), and their composite materials have been examined for thermoelectric and storage performance (Tatrari *et al.*).^11–13^ Both pure and heterostructured TMOs are widely used in energy storage due to their compositional tunability and electrochemical activity. S. Kumar, M. Yin and Z. Lei have demonstrated that pure oxides such as NiCo_2_O_4_ and MnO_2_, as well as layered or two-dimensional (2D) TMOs, show great promise in applications such as supercapacitors, batteries, water splitting and oxygen electrocatalysis.^14–16^ However, their performance is often limited by factors such as low conductivity, a small number of active sites, charge recombination, and loss of stability during operation. Consequently, recent work has focused on defect engineering, heteroatom doping and controlling morphology, particularly the construction of heterostructures, to enhance charge transfer, increase the number of active sites and stabilise redox cycling (A. Vazhayil *et al.*).^17^

Forming heterojunctions between two or more metal oxides accelerates electron transfer and creates abundant interfacial active sites, consistently outperforming single-phase counterparts.

Heterostructured systems, such as Co_3_O_4_–CuO, ZnO/NiO, Co_3_O_4_/CeO_2_, NiO/CeO_2_, ZnWO_4_@ZnO, TiO_2_/rGO, ZrO_2_/NiO and CuS/CdFe_2_O_4_/CNF, generally outperform single oxides because interfacial synergy accelerates electron transport and increases the electroactive surface area. This behaviour has been observed by several research groups, including those of N. R. Reddy, C. Du and Y. Zhang, and has been shown to improve the storage and catalytic kinetics.^18–22^ Representative results include 2025.6 F g^−1^ for oxygen-deficient ZnO/NiO supercapacitor electrodes, 395 F g^−1^ for Co_3_O_4_–CuO, 217 mAh g^−1^ at 10 °C over 5000 cycles for TiO_2_/rGO sodium storage, 432 C g^−1^ for Zr/Ni oxide electrodes, and low hydrogen evolution reaction (HER) overpotentials of 88–265 mV for oxide/CeO_2_ heterostructures. Meanwhile, Vattikuti *et al.*, B. Li and D. Sahu have conducted studies on related ternary photocatalytic heterojunctions such as MoS_2_-BN/TiO_2_ and g-C_3_N_4_@ZnO:MoS_2_, which enhance hydrogen evolution, dye degradation and photoinduced charge separation.^23–25^ Furthermore, TMO composites comprising g-C_3_N_4_, MoS_2_ and MXene-derived phases are being developed for use in photocatalysis-assisted supercapacitors and Z-scheme water splitting. The H_2_ production rate of MoS_2_-BN/TiO_2_ ternary heterostructures, for example, is 2.6 mmol g^−1^ h^−1^, achieved *via* synergistic interfacial charge separation (D. Sahu *et al.*, S. A. Ali *et al.*).^26,27^

As demonstrated by Ahmed *et al.* and reviewed in detail by Chauhan *et al.*, cobaltite (Co_3_O_4_) spinel-based nanocomposites are a versatile material with a wide range of applications, including in environmental remediation, electrocatalysis, and energy storage *via* supercapacitors. This spinel is active under visible light and exhibits two accessible oxidation states (Co^2+^/Co^3+^), as well as having teneable nanostructures.^28,29^ As detailed in the reviews by Zhao and Ma, as well as by Nashim *et al.*, the primary design strategy for these Co_3_O_4_-based nanocomposites is to form heterojunctions or hybrid interfaces with various inorganic materials, such as metal oxides, carbon-based materials, chalcogenides, conductive metals, and dopants. This improves charge transfer, suppresses electron–hole recombination, and increases the number of oxygen vacancies, thereby exposing more catalytically active surface sites.^29–31^

Since the synthesis route significantly influences interfacial contact, crystallinity, porosity and electrochemical behaviour, hydrothermal synthesis at a relatively low temperature is commonly employed to produce cobaltite-based heterostructured materials. This ensures control over phase purity, composition, particle growth and morphology. The resulting nanocomposites, such as CeO_2_–Co_3_O_4_, Co_3_O_4_–CoFe_2_O_4_ and ZnSe/Co_3_O_4_, feature nanostructures comprising pure phases.^32–34^

Other commonly used synthesis routes include the sol–gel process for Co_3_O_4_/Ag/CuO composites; mechanochemical milling for Co_3_O_4_ on nitrogen-doped multiwalled carbon nanotubes; and thermal conversion from metal–organic frameworks (MOFs) for iron-doped Co_3_O_4_.^35–37^

In photocatalytic and photoelectrocatalytic water treatment, Co_3_O_4_-based materials are used to degrade dyes, antibiotics, phenols, and hexavalent chromium (Cr(vi)). Studies by Zaho and Chauhan confirm that the performance of these materials generally improves when heterostructures are formed, thereby enhancing light absorption and the efficiency of photogenerated carriers.^29,30^ For istance, combining the bandgap of cobaltite (Co_3_O_4_, 1.5 eV) with that of cadmium aluminium oxide (CdAl_2_O_4_, 3.66 eV) creates a cobaltite/cadmium aluminium oxide nanocomposite with a bandgap of 2.4 eV. This nanocomposite material exhibits enhanced visible light absorption and reduced recombination, as well as dye removal efficiencies of 91% for Direct Blue and 86% for Basic Yellow (Z. Araei *et al.*).^38^ Similarly, ZnSe/Co_3_O_4_ heterostructured materials achieved a 96% degradation rate for Congo red due to synergistic band alignment, which suppressed electron–hole recombination. Additionally, C-type Co_3_O_4_/Bi_4_O_5_I_2_/Bi_5_O_7_ composites reduced recombination and enabled the high mineralisation of *p*-nitrophenol and Brilliant Black (Malefane *et al.*).^34,39^

Furthermore, Yousefi *et al.* have demonstrated that Co/Co_3_O_4_ nanocomposites can achieve 93% decolourisation of Acid Red 151, as well as exhibiting measurable antibacterial activity against clinical pathogens. The environmental significance of cobalt oxide-based composites extends well beyond their ability to remove pollutants using light. In the field of oxygen electrocatalysis, for instance, the performance of Co_3_O_4_-based nanocomposites hinges on their surface structure, electrical conductivity and ability to interact with one another.^40^

The performance of graphene-supported Co_3_O_4_ nanocrystals in the oxygen evolution reaction (OER) can vary considerably depending on the surface's properties. The presence of Co^2+^ ions on the surface is crucial to this reaction. For instance, Co_3_O_4_ deposited on a nitrogen-doped reduced graphene oxide (NRGO) surface exhibits morphologies comprising numerous Co^3+^ sites, which absorb oxygen effectively and promote evolution and reduction reactions (Xiao and Han).^41,42^

Furthermore, the development of a highly active bifunctional electrocatalyst using Co_3_O_4_ on carbon nanotubes for oxygen reduction and evolution, as demonstrated by Ahmed *et al.*, has shown superior performance to other materials such as platinum/carbon (Pt/C) and ruthenium oxide (RuO_2_). This is due to the electrocatalyst's porous structure and the strong interaction between the Co_3_O_4_ and the carbon nanotubes, which increases the number of active sites and facilitates current flow through the material.^28^

In the field of electrochemical energy storage, pure Co_3_O_4_ shows great potential. Several studies have confirmed that pH-optimised hydrothermal cobaltite nanoparticles demonstrate a capacity of 1195 F g^−1^ in a three-electrode configuration and 870.6 F g^−1^ in a symmetric device. Coprecipitated Co_3_O_4_ nanorods have also shown excellent performance, achieving 373.84 F g^−1^ with 92% capacity retention after 1000 cycles thanks to their high surface-to-volume ratio, which promotes redox activity (Han, Babu, and Karthikeyan).^42–44^

Additionally, the Co_3_O_4_/Ag/CuO composite material exhibited impressive values of 1697 F g^−1^ and 99.62 Wh kg^−1^, remaining stable over 10 000 cycles because of the nanoporous CuO network. Even higher values were obtained when the composition and electrolyte were designed together. Co_3_O_4_ derived from an iron-doped metal–organic framework (MOF) delivered approximately 2135 F g^−1^, and an asymmetric device achieved 51.99 Wh kg^−1^ with approximately 89% retention over 10 000 cycles. Furthermore, the capacity of ‘blade-like’ Co_3_O_4_ on nickel foam increased from 1560–6580 F g^−1^ following optimisation using an electrolyte containing a redox additive (Shah and Dhakal).^45,46^

It is important to note that carbon- and Co_3_O_4_-based composites remain competitive options for device fabrication. For example, Co_3_O_4_ spinel heterostructured with nitrogen-doped multi-walled carbon nanotubes, known as Co_3_O_4_-NMWCNT, have achieved a capacitance of 202 F g^−1^ and an energy density of 25 Wh kg^−1^. This demonstrates that conductive carbon structures can enhance electron transport, even when the capacity increase is smaller than in multicomponent oxide systems (Dhakal *et al.*).^46^

Overall, studies of Co_3_O_4_ in combination with other materials demonstrate that it is most effective when used in this way. The properties of this platform are determined by its morphology, which is dependent on the synthesis process, the exposure of its crystal planes, its interfacial bonds, and the synergy between its components. This enables a wide range of applications in pollutant degradation, oxygen electrocatalysis, and high-performance supercapacitors.

Although the catalytic and environmental properties of Co_3_O_4_-based composite materials have been extensively studied, their semiconducting properties and conduction mechanisms remain largely unexplored. As part of the strategy to develop ‘smart’ multifunctional materials, this study examines the catalytic properties of Co_3_O_4_-based composite materials in two ways: by degrading organic dyes and by using them in electrochemical energy storage. The effectiveness of these materials as electrodes comprising metal oxides coupled with Co_3_O_4_ is also evaluated.

## Experimental section and methods

### Materials

The following materials were used as received without further purification: cobalt nitrate hexahydrate (99%, Aldrich), titanium dioxide (99%, Aldrich), zinc oxide (99%, Aldrich), copper nitrate trihydrate Cu(NO_3_)_2_·3H_2_O(99.9%, Aldrich), polyvinylpyrrolidone PVP (average molecular weight ∼55 000 g mol^−1^, Aldrich), urea CH_4_N_2_O (98%, Aldrich), ethylene glycol C_2_H_6_O_2_(99%, Aldrich) hydrochloric acid HCl (37%, Aldrich) and sodium hydroxide pellets NaOH (98%, Aldrich). Deionised water was used for all synthesis and washing steps, while ethanol was only used for washing.

### Synthesis procedure

Co_3_O_4_-based nanocomposites were obtained *via* a one-pot hydrothermal process. In the first stage, the reagents were carefully dissolved in the minimum possible volume of water, in the presence of 5 ml of ethylene glycol, 0.1 g of PVP and 10^−2^ mol of urea. The mixture was then stirred mechanically for two hours to ensure complete homogenisation. For the ZnO and TiO_2_ reagents, these were first dissolved in hydrochloric acid to form aqueous solutions of ZnCl_2_ and TiCl_4_, which were then added to the cobalt nitrate solution. The solutions were adjusted to pH 11 with 3 M NaOH to ensure the formation of metal hydroxide. This mixture was transferred to an autoclave and acetylated at 180 °C for 12 hours.

The products obtained were filtered and washed several times with distilled water and ethanol, then dried in an oven at 80 °C for 12 hours. The products then underwent a first heat treatment at 450 °C for four hours to remove any residual impurities (CO_2_, NH_3_ and H_2_O). They were then annealed at 600 °C for seven hours to obtain the desired phases.

### Characterisation techniques

XRD analysis was conducted using a Bruker D8 Advance diffractometer with CuKα radiation (*λ* = 1.5406 Å). X-ray diffraction data were collected with a step size of 0.02°, using a LynxEye detector set to a counting time of 12 seconds and a variation in 2*θ* between 10° and 70°.

FTIR spectra were recorded using a PerkinElmer UATR II FT-IR spectrometer in the frequency range of 1500–450 cm^−1^.

HRTEM (Tecnai F30 G2, FEI, Hillsboro, Oregon, USA) was used to analyse the particles on a nanometre scale.

XPS (VG ESCALA B220i–XL, Thermo Scientific, Surrey, UK) was used to analyse the surface properties of the samples with an Al Kα (*hν* = 1486.6 eV) source at a residual gas pressure below 10^−8^ Pa, and all the binding energies were referenced to the C 1s peak at 284.8 eV of the surface adventitious carbon.

The textural properties of the prepared catalyst were investigated using an ASAP2020 Micromeritics analyser *via* nitrogen adsorption/desorption. To ensure the elimination of all adsorbed gases, the samples were degassed at 200 °C under 8 µmHg for 720 minutes. Isotherms were measured at 77 K over a *P*/*P*_0_ range of 10^−5^ to 1. The specific surface area (*S*_BET_) was calculated using the BET model and the pore distribution was determined using the BJH model.

Spectroscopic measurements of diffuse reflectance in the 200–800 nm range were used to investigate the optical properties of pure Co_3_O_4_ and Co_3_O_4_-based nanocomposite materials.

The resulting spectra were fitted to the Kubelka–Munk equation. expressed as follows:^47^1
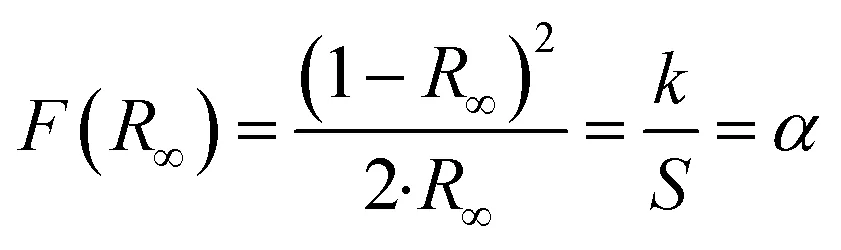
In the following equation, *F*(*R*_∞_) is equivalent to the Kubelka–Munk function (*i.e.*, the inverse of the absorption coefficient), *R*_∞_ is the absolute diffuse reflectance of an infinitely thick sample relative to a reference that does not absorb the light, *k* is the absorption coefficient, *S* is the scattering coefficient, and *α* is the material's absorption coefficient.

Optical transitions in semiconductors can be attributed to direct or indirect mechanisms. The optical bandgap (*E*_g_) is defined as the energy required to excite electrons from the valence band to the conduction band. The Tauc plot method was used to determine *E*_g_, with the following relation being employed: the expression for the transition is given by the following formula:

2 *F*(*R*_∞_)*hν* = *A*·(*hν* − *E*_g_)^*n*^

where *h* is Planck's constant and *A* is an additional constant. The photon energy, *hν*, is expressed in eV, whereas *E*_g_ indicates the bandgap energy (eV). The transition type is denoted by *n*, which can be 2, 3, 1/2 or 3/2 according to the following classification: indirect allowed, indirect forbidden, direct allowed or direct forbidden. The bandgap was obtained by extrapolating the linear region of [*F*(*R*_∞_)*hν*]^1/*n*^*versus hν* to zero.

The incident photon energy was calculated using the following formula:

3 *hν* = 1240/*λ*

The photocatalytic performance of the synthesised nanocomposite materials was evaluated by monitoring the degradation of RhB and MB when exposed to solar and UV light. The catalyst loading used was 1.5 g L^−1^, and the system was first equilibrated in the dark for 60 minutes to establish adsorption–desorption equilibrium. During irradiation, samples were collected at 5–135-minutes intervals, then centrifuged and filtered prior to UV-Vis spectrophotometric analysis (*λ* = 550 nm for RhB and *λ* = 663 nm for MB). Degradation efficiency was calculated using the Beer–Lambert law. The reaction kinetics were analysed *via* a pseudo-first-order model to determine the degradation rate constant.

EIS measurements were performed using an Agilent 4284A LCR meter, which has a frequency range of 20 Hz to 1 MHz and an applied AC voltage of 50 mV. The experiments employed a two-electrode, open-circuit configuration with silver paste electrodes and platinum wire current collectors. Temperature-dependent studies were performed using a Toky intelligent temperature controller ranging from 25 °C to 600 °C. Electric conductivity (*σ*) was calculated using the formula:^47^4
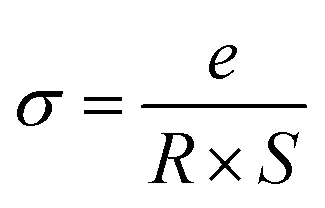
where *e* represents the pellet thickness, *R* denotes the bulk resistance derived from impedance spectra and *S* represents the electrode surface area.

## Results and discussion

### XRD analysis

Fig. 1 shows the powder XRD diffractograms of the synthesised nanocomposites. All diffraction peaks were compared with reference files from the ICDD database. Co_3_O_4_ (ICDD file no. 00-009-0418 (peaks at 2*θ*: 19.09°, 31.38°, 36.88°, 38.72°, 44.88°, 55.80°, 59.65°, 65.44°), and ZnO (ICDD file no. 00-001-1136; peaks at 2*θ*: 31.87°, 34.51°, 47.63°, 56.76°, 63.23°, 66.50°, 68.09° and 69.31°), TiO_2_ (ICDD file no. 00-001-0562; peaks at 2*θ*: 25.35°, 38.04°, 48.26°, 53.96°, 55.18°, 62.79°, 68.92°) and CuO (ICDD file no. 00-001-1117; peaks at 2*θ*: 35.72°, 39.01°, 48.99°, 53.67°, 58.50°, 61.78°, 66.24°, 68.15°). The diffractograms reveal distinctive peaks associated with the cubic spinel phase of Co_3_O_4_ (*Fd*3̄*m* space group) and the respective MO_*x*_ oxide phase. This confirms that the materials under study comprise two distinct heterojunction phases rather than resulting from substitution or doping. However, it should be noted that the intensity of the Co_3_O_4_ peaks varies considerably within each heterojunction, depending on the nature of the oxidised metal. This behaviour may be due to mass dilution and signal absorption (masking) by the outer phase (TiO_2_, ZnO or CuO). Furthermore, lattice misalignment at the interface generates microdeformations that broaden the peaks and reduce their height. Additionally, phenomena such as preferential orientation and ionic interdiffusion can alter the crystal structure factor locally, thereby directly affecting the response of the diffraction plane, as demonstrated by Shi and Han.^48,49^

**Fig. 1:**
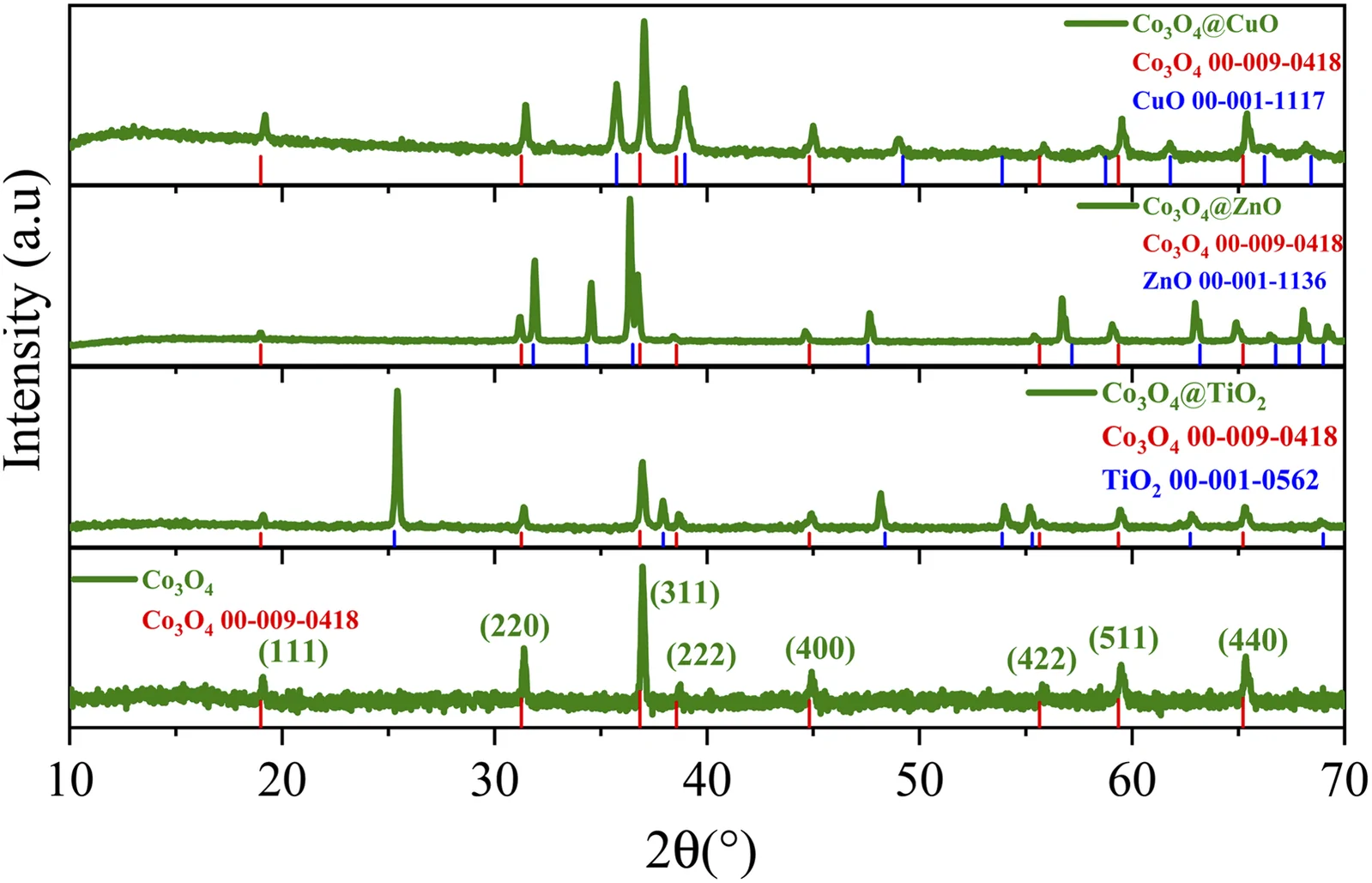
Powder XRD patterns of the Co_3_O_4_@M-O_*x*_ nanocomposites.

### Rietveld structural refinement

This study uses the Rietveld method to refine the structures of the obtained nanocomposites. Fig. 2 depicts the refined profiles of the synthesized materials, and Table 1 presents the lattice parameters and adjustment factors. A detailed analysis of the obtained values confirms the high crystallinity of the synthesized materials and their proportions within each phase.^50,51^

**Fig. 2:**
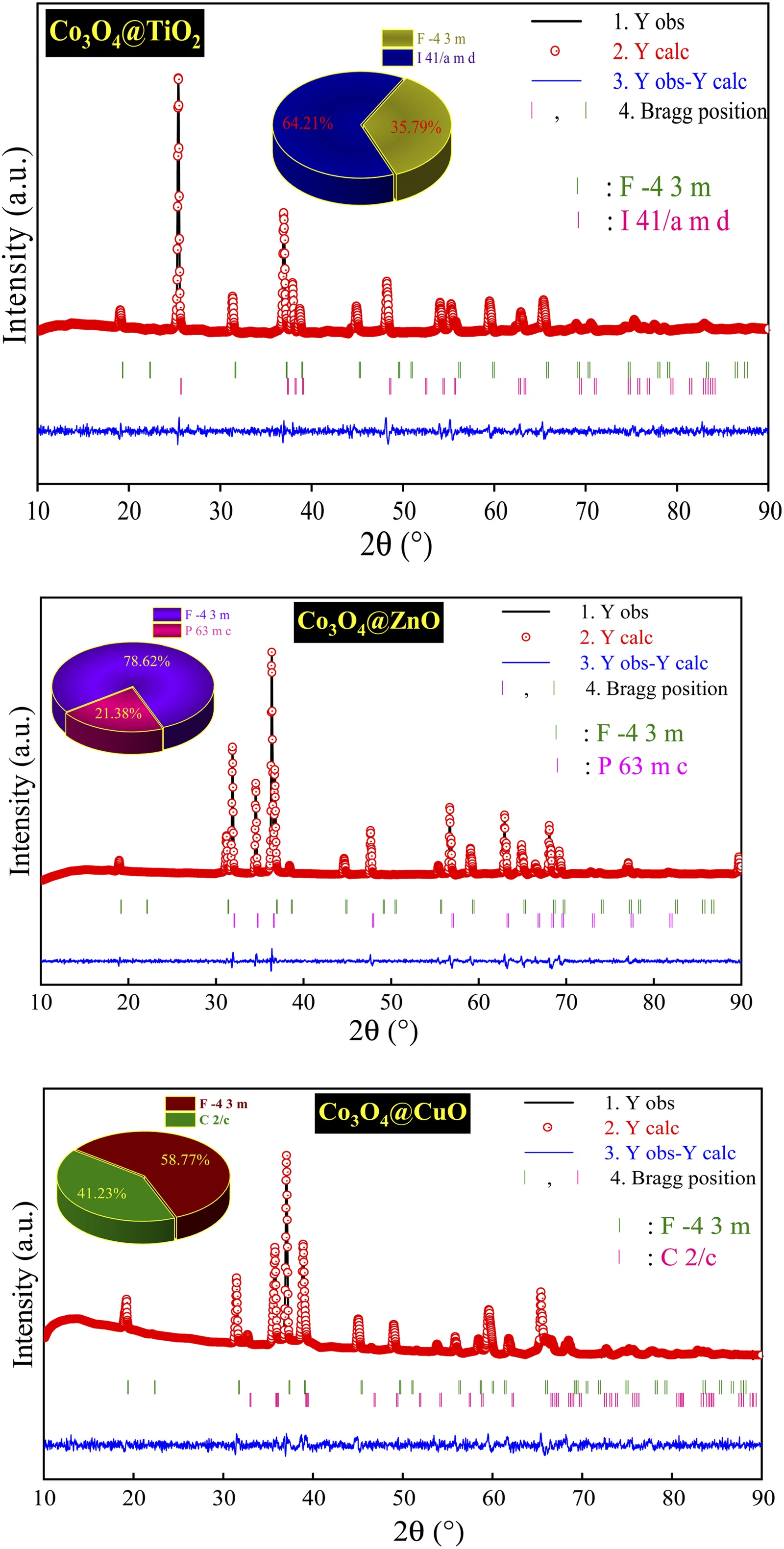
Rietveld refinement patterns recorded at room temperature of the synthesized samples. In these figures, calculated and observed profiles are included. The difference between these profiles is shown underneath.

**Table 1:** Refined cell parameters and GOF for the nanocomposite materials

	Co_3_O_4_@CuO		Co_3_O_4_@TiO_2_		Co_3_O_4_@ZnO	
	Co_3_O_4_	CuO	Co_3_O_4_	TiO_2_	Co_3_O_4_	ZnO
S.G	*F*4̄3*m*	*C*2/*c*	*F*4̄3*m*	*I*4_1_/*amd*	*F*4̄3*m*	*P*6*3mc*
*a* (Å)	8.049	4.667	8.069	3.771	8.114	3.241
*b* (Å)	8.049	3.405	8.069	3.771	8.114	3.241
*c* (Å)	8.049	5.115	8.069	9.490	8.114	5.193
*V* (Å^3^)	521.593	80.22	525.47	135.00	534.33	47.265
*α* (°)	90.00	90.00	90.00	90.00	90.00	90.00
*β* (°)	90.00	99.37	90.00	90.00	90.00	90.00
*γ* (°)	90.00	90.00	90.00	90.00	90.00	120.00
*F*4̄3*m* (%)	58.77	—	35.79	—	78.62	—
*C*2/*c* (%)	—	41.23	—	—	—	—
*I*4_1_/*amd* (%)	—	—	—	64.21	—	—
*P*6_3_*mc* (%)	—	—	—	—	—	21.38
*R*_wp_ (%)	3.65	3.78	2.52	2.70	3.75	3.26
*R*_b_(%)	3.37	3.42	2.01	2.00	1.64	1.63
*R*_p_ (%)	3.13	3.21	1.70	1.80	1.97	2.27
*χ* ^2^	1.77	1.97	1.57	1.81	2.01	1.93

Notably, variations in the percentage of the Co_3_O_4_ phase in each material shed light on the morphological architecture and electronic charge transfer mechanisms of these nanostructures. In the case of Co_3_O_4_@ZnO (78.62%), the strong predominance of the cobalt phase confirms the presence of a classic heterojunction structure.

In this structure, a large Co_3_O_4_ semiconductor (p-type) is decorated by a thin ZnO semiconductor (n-type), forming a p–n heterojunction with a high potential barrier. Conversely, the low cobalt content of Co_3_O_4_@TiO_2_ (35.97%) indicates an inverted structure with an ultrathin Co_3_O_4_ layer on the surface of the TiO_2_ (n-type) oxide.

This configuration is ideal for a direct S- or Z-scheme mechanism, in which photogenerated electrons from the TiO_2_ conduction band recombine with holes in the Co_3_O_4_ valence band to maximise redox capacity. The nearly equal proportions of Co_3_O_4_ and CuO in Co_3_O_4_@CuO (58.77%) promote the formation of a p–p heterojunction, where both oxides are p-type semiconductors. Several studies, including those by Liu, Iqbal and Mei, confirm that this structure is highly effective for energy band alignment and rapid hole extraction.^52–54^

### XPS analysis

Fig. 3 shows the XPS results for Co_3_O_4_ and Co_3_O_4_@TiO_2_. The XPS spectra of Co_3_O_4_@ZnO and Co_3_O_4_@CuO can be found in Fig. S1. The broad Co_3_O_4_ spectrum confirms the presence of cobalt and oxygen as the primary elemental constituents. The wide range reveals distinct photoelectron and Auger peaks characteristic of these elements. The most prominent features are the intense Co 2p doublet and the O 1 s signal. The Co 2p spectrum shows two main spin-orbital split peaks with maximum intensities of approximately 780 eV and 794 eV for Co 2p_3/2_ and Co 2p_1/2_, respectively. Through spectral deconvolution, these envelopes can be resolved into multiple components that correspond to different cobalt cation sites within the spinel structure. Higher binding energy contributions are assigned to octahedrally coordinated Co^3+^ species, while lower binding energy components are assigned to tetrahedrally coordinated Co^2+^ species. The asymmetric O 1s core-level spectrum, centred near 528 eV, is typically fitted with three components: a dominant peak at the lowest binding energy, which corresponds to lattice oxygen (O^2−^) in the oxide structure; a second component, which is associated with oxygen vacancies or defect sites and is located at a slightly higher energy; and a third component, which arises from surface-adsorbed oxygen-containing impurities or hydroxyl groups (Rana *et al.*, Reda *et al.*, Ungeheuer *et al.*).^55–57^

**Fig. 3:**
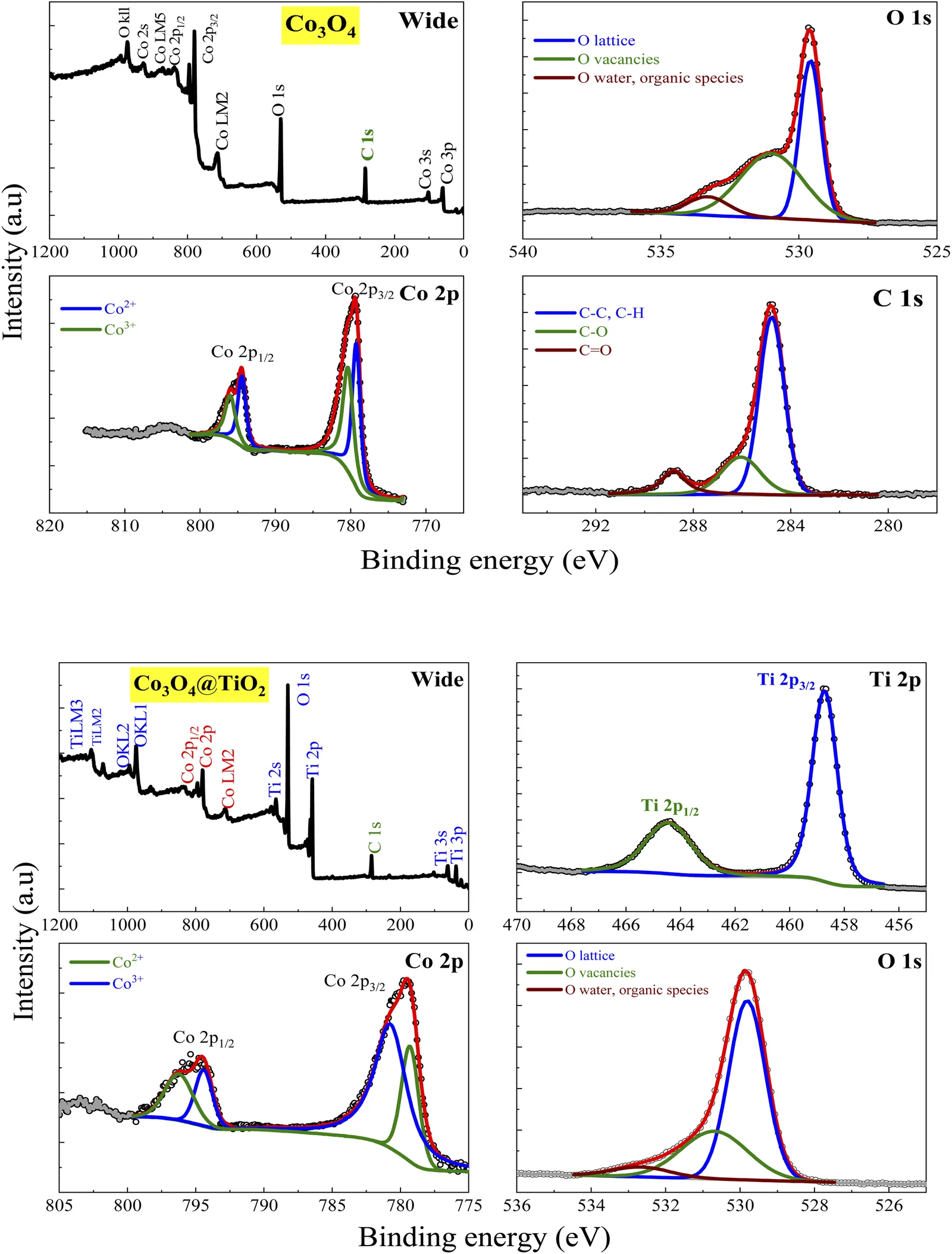
XPS spectra of pure Co_3_O_4_ and Co_3_O_4_@TiO_2_ nanocomposite materials.

The XPS spectrum of the Co_3_O_4_@TiO_2_ material provides compelling evidence that a successful binary nanocomposite has formed. The wide-scan spectrum of this material retains all the Co_3_O_4_ peaks and introduces the definitive Ti 2p doublet, thus validating the heterojunction architecture. Specifically, the Ti 2p spectrum separates into two distinct spin–orbit components, with the Ti 2p_3/2_ and Ti 2p_1/2_ peaks centred at approximately 458 eV and 464 eV, respectively. This characteristic splitting, with a spin–orbit separation of approximately 6 eV, is unequivocally attributed to the Ti^4+^ oxidation state within the tetragonal TiO_2_ lattice, corresponding to Ti–O bonds in the oxide framework. Notably, the Co 2p_3/2_ and Co 2p_1/2_ maxima in the Co_3_O_4_@TiO_2_ XPS spectrum remain at their original positions of 780 eV and 794 eV, respectively. The deconvoluted subpeaks indicate octahedral and tetrahedral sites of Co^3+^ and Co^2+^ from the pure Co_3_O_4_ phase. The absence of any significant chemical shift in the core-level binding energies for either cobalt or titanium indicates that substantial electron transfer or intermixing at the interface would not affect their oxidation states, as demonstrated by Reda and Krishnan.^56,58^

### TEM and HRTEM analysis

Fig. 4a and e show transmission electron microscopy (TEM) images of Co_3_O_4_ and Co_3_O_4_@TiO_2_, respectively. These images reveal spherical particles of various sizes consisting of Co_3_O_4_ and TiO_2_ at the nanometre scale. TEM images of the Co_3_O_4_@ZnO and Co_3_O_4_@CuO nanocomposite materials are shown in Fig. S2. The size distribution diagrams (Fig. 4b and f) show that the mean diameter of the particles is 90.7 nm for Co_3_O_4_ and 76.1 nm for TiO_2_. The HRTEM images (Fig. 4c and g) clearly show the lattice fringes of the spinel cobalt and titanium oxide materials, suggesting that the heterostructures are highly crystalline.

**Fig. 4:**
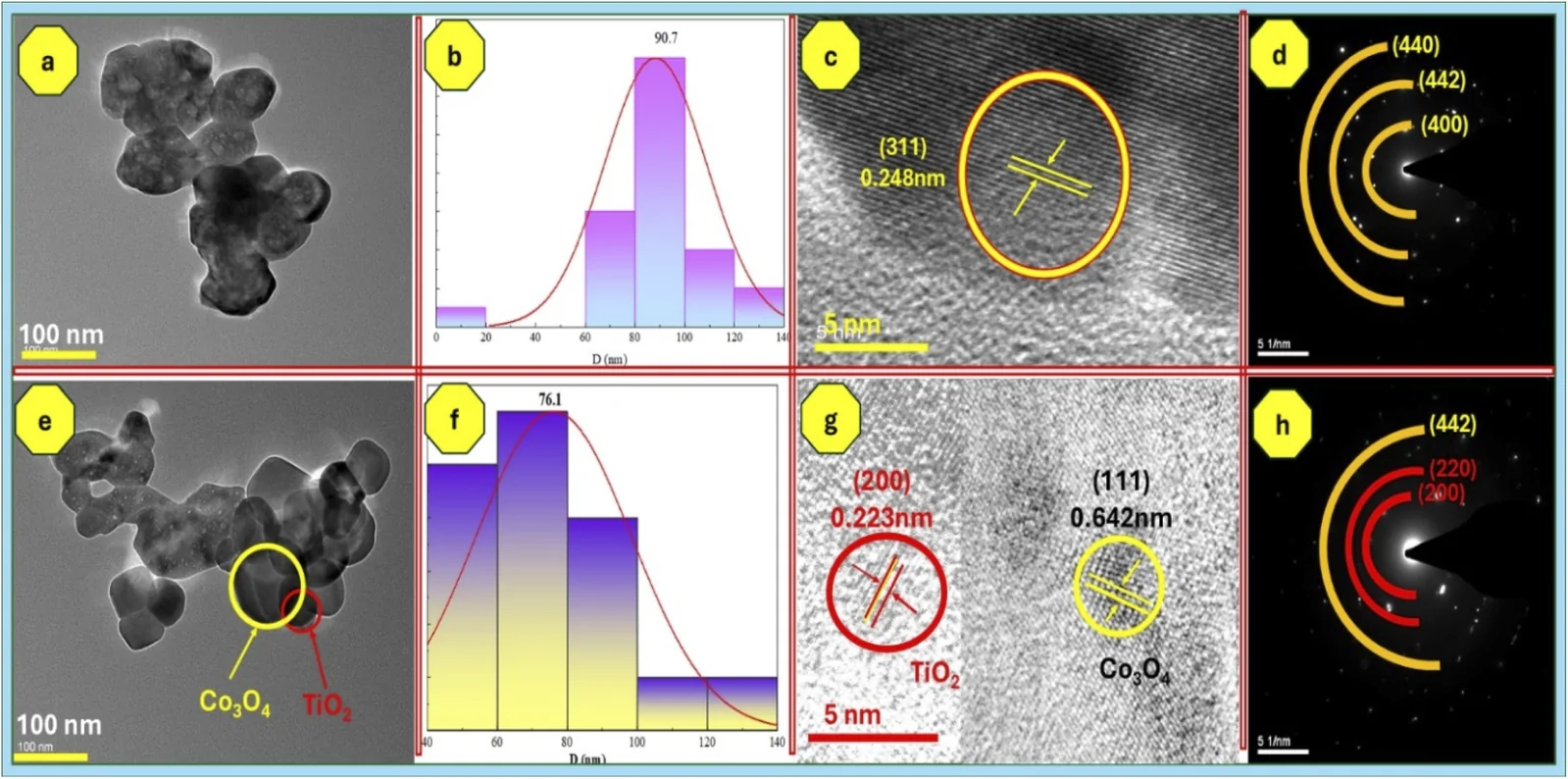
Co_3_O_4_ sample: TEM images (a), distribution diagram (b), HRTEM images (c), SAED pattern (d). Co_3_O_4_@TiO_2_ sample: TEM images (e), distribution diagram (f), HRTEM images (g), SAED pattern (h).

The interplanar distances of 0.248 nm and 0.642 nm correspond to the (311) and (111) planes of cubic Co_3_O_4_, respectively, as shown in figures.^59,60^ The distance of 0.223 nm corresponds to the (200) plane of body-centred cubic TiO_2_, as previously confirmed by XRD analysis. Furthermore, continuity of the diffraction peaks was observed, as well as a distinct interface between the Co_3_O_4_ and TiO_2_ layers, suggesting the formation of a p–n heterojunction within the Co_3_O_4_/TiO_2_ heterostructures. This is also confirmed by the selected area electron diffraction (SAED) diagrams in Fig. 4d and h. The presence of a highly crystalline, preferentially oriented single-crystal cubic Co_3_O_4_ nanostructure is confirmed by the observation of distinct diffraction spots rather than concentric rings. According to Reda *et al.*, these spots can be indexed to the spinel-type cubic phase of Co_3_O_4_ and the body-centered cubic phase of TiO_2_.^56^

### FTIR spectroscopy analysis

Fig. 5 shows the FTIR spectra Co_3_O_4_ and its nanocomposites with zinc oxide (ZnO), titanium dioxide (TiO_2_) and copper oxide (CuO). These spectra exhibit distinct absorption bands that provide insight into the structural and compositional characteristics of the materials. The spectra confirm the purity of the obtained phases and indicate the presence of bands associated with different types of bonds. Consequently, the nature of the metal–oxygen and Co–O absorption bands in each nanocomposite is significantly influenced by the type of heterojunction formed. The FTIR spectrum of the Co_3_O_4_ material generally reveals absorption peaks associated with the cubic spinel structure, with characteristic bands at 545 and 651 cm^−1^, which are specific to the spinel oxide phase (Liaqat *et al.*).^61^

**Fig. 5:**
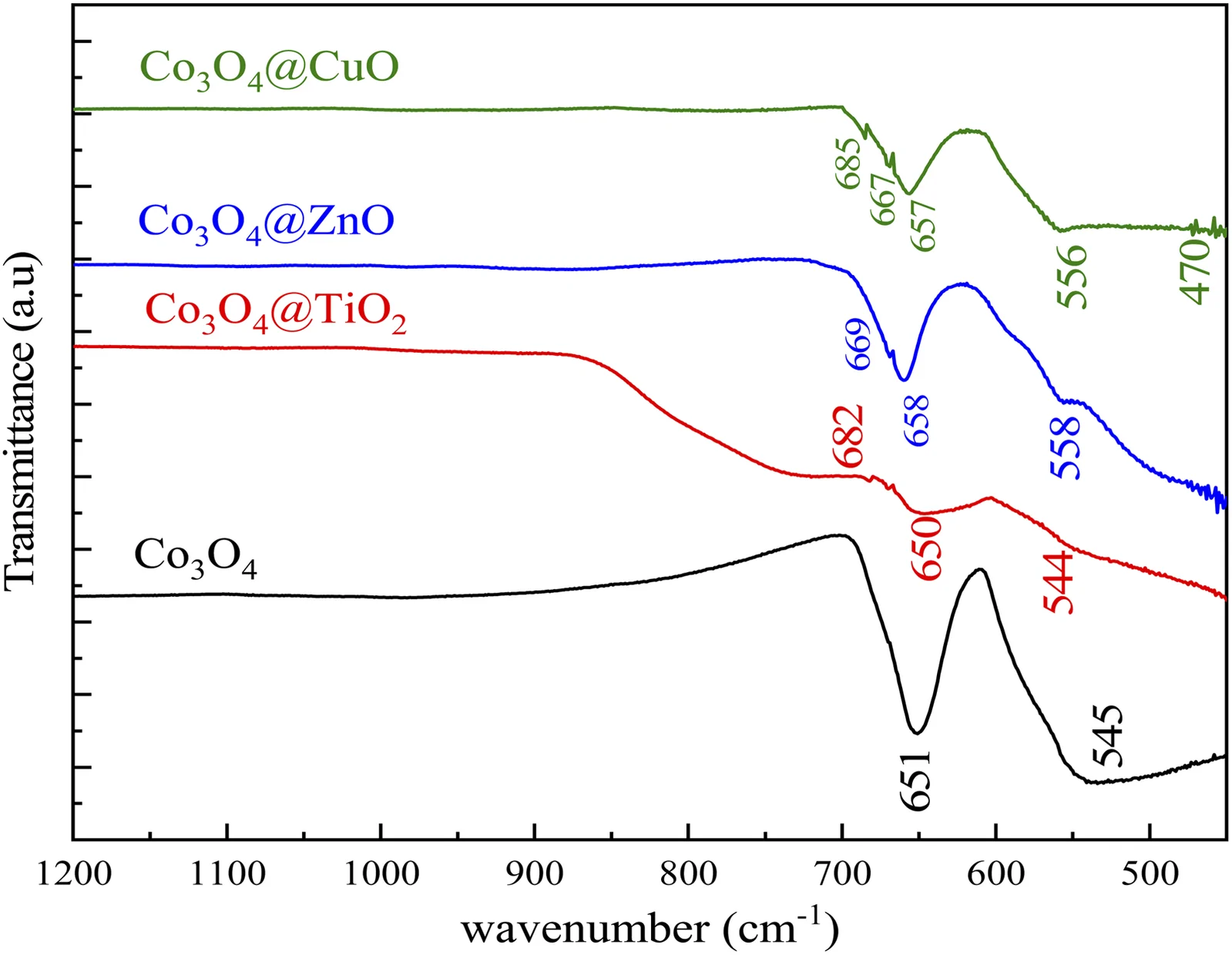
FTIR spectra of the Co_3_O_4_-based nanomaterials.

In this structure, Co^2+^ and Co^3+^ ions are directly bonded to oxygen ions *via* Co–O bonds (Shahid and Reikowski).^62,63^

Moreover, forming a heterojunction with ZnO can lead to the appearance of a band attributed to the Zn^2+^–O^2-^ bond at 558 cm^−1^. This reflects changes in the electronic environment and the formation of new interfaces, increasing the mobility of charge carriers (Alhashem *et al.*).^64^

The FTIR spectrum of the Co_3_O_4_@TiO_2_ nanocomposite indicates the presence of vibrational bands from Co_3_O_4_, as well as overlapping bands attributed to the Ti^4+^–O^2-^ stretching vibration at 682 cm^−1^. This interaction may alter the electronic properties of the nanocomposite, thereby enhancing its photocatalytic activity (Shi *et al.*).^48^

The FTIR spectrum of the Co_3_O_4_@CuO nanocomposite is more complex, displaying characteristic absorption bands for both pure Co_3_O_4_ and CuO. The vibrational band at 470 cm^−1^ is associated with Cu–O stretching vibrations, and the band between 657 and 685 cm^−1^ corresponds to vibrational modes of Co_3_O_4_. These results suggest a successful junction between the two components. This behaviour enhances the nanocomposite's catalytic activity, particularly in the photoreduction of rhodamine 6G textile effluent catalysed by annealed metamorphic nano-CuO, due to the synergistic effects of the two oxides as demonstrated by Sheng.^65^

### BET and surface distribution

Fig. 6 and Table 2 show the BET analysis of the various Co_3_O_4_-based nanocomposites. The nitrogen adsorption–desorption isotherms of the nanocomposites exhibit type IV profiles with H3 hysteresis, which is indicative of mesoporous solid materials with interconnected, non-uniform, slit-shaped mesopores (Souemti *et al.*).^47^ The textural properties reveal that Co_3_O_4_@TiO_2_ exhibits the greatest specific surface area (5.116 m^2^ g^−1^) and pore volume (0.038 cm^3^ g^−1^), demonstrating enhanced porosity compared to pure Co_3_O_4_ (3.488 m^2^ g^−1^ and 0.025 cm^3^ g^−1^), respectively). In contrast, Co_3_O_4_@ZnO exhibits the lowest specific surface area (2.985 m^2^ g^−1^) and pore volume (0.008 cm^3^ g^−1^), despite possessing the largest mean pore diameter (34.5 nm). This suggests significant pore blockage or densification of the structure. Furthermore, Co_3_O_4_@CuO exhibits intermediate values (3.684 m^2^ g^−1^ and 0.015 cm^3^ g^−1^), reflecting a less porous but more compact architecture. This is likely due to different interfacial interactions and particle arrangements, given its smaller pore size of 26.8 nm. These findings directly influence a material's potential catalytic or adsorption performance.

**Fig. 6:**
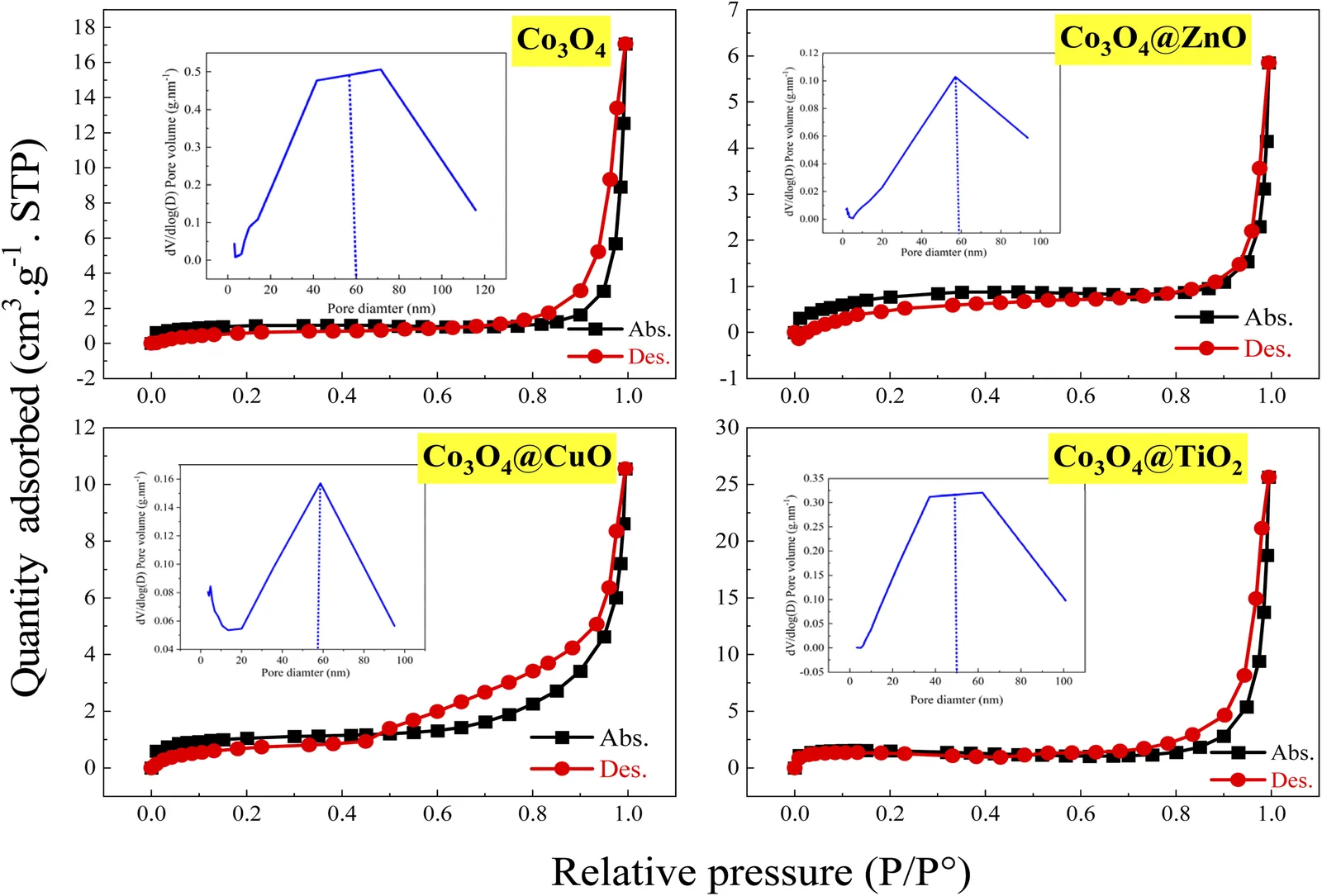
N2 adsorption and desorption isotherm linear plots and BJH desorption pore distributions of the Co_3_O_4_-based nanocomposites.

**Table 2:** BET characteristics of Co3O4-based nanocomposite materials

	*S*_BET_ (m^2^ g^−1^)	*D*_p_ (nm)	*V*_p_ (cm^3^ g^−1^)
Co_3_O_4_	3.488	30.261	0.025
Co_3_O_4_@TiO_2_	5.116	31.975	0.038
Co_3_O_4_@ZnO	2.985	34.515	0.008
Co_3_O_4_@CuO	3.684	26.829	0.015

Following the methodology established by Chu *et al.*, the *in situ* images in each figure depict the average pore diameter. The fact that the pore diameter remains strictly unchanged at 60 nm for the pure Co_3_O_4_, Co_3_O_4_@ZnO and Co_3_O_4_@CuO samples indicates that the ZnO and CuO phases were deposited or grew selectively on the outer surface of the matrix. In contrast, the decrease in diameter to 55 nm observed for the Co_3_O_4_@TiO_2_ system specifically reflects internal encapsulation or partial channel blockage (‘pore clogging’). The TiO_2_ precursor penetrated deeper into the porous Co_3_O_4_ network, crystallised along the inner walls and reduced the available free space by 5 nm. This result confirms that the close core–shell interaction extends into the internal porosity of the material, as demonstrated by HRTEM analysis.^66^

### UV–visible diffuse reflectance spectroscopy (DRS) analysis

Fig. 7 shows the Tauc plots for the synthesised nanocomposite materials. The bandgap energies were obtained by taking the tangent to the curves at the *x*-axis, which suggests an indirect transition (*n* = 1/2).^47^ The obtained bandgap energy values are summarised in Table 3.

**Fig. 7:**
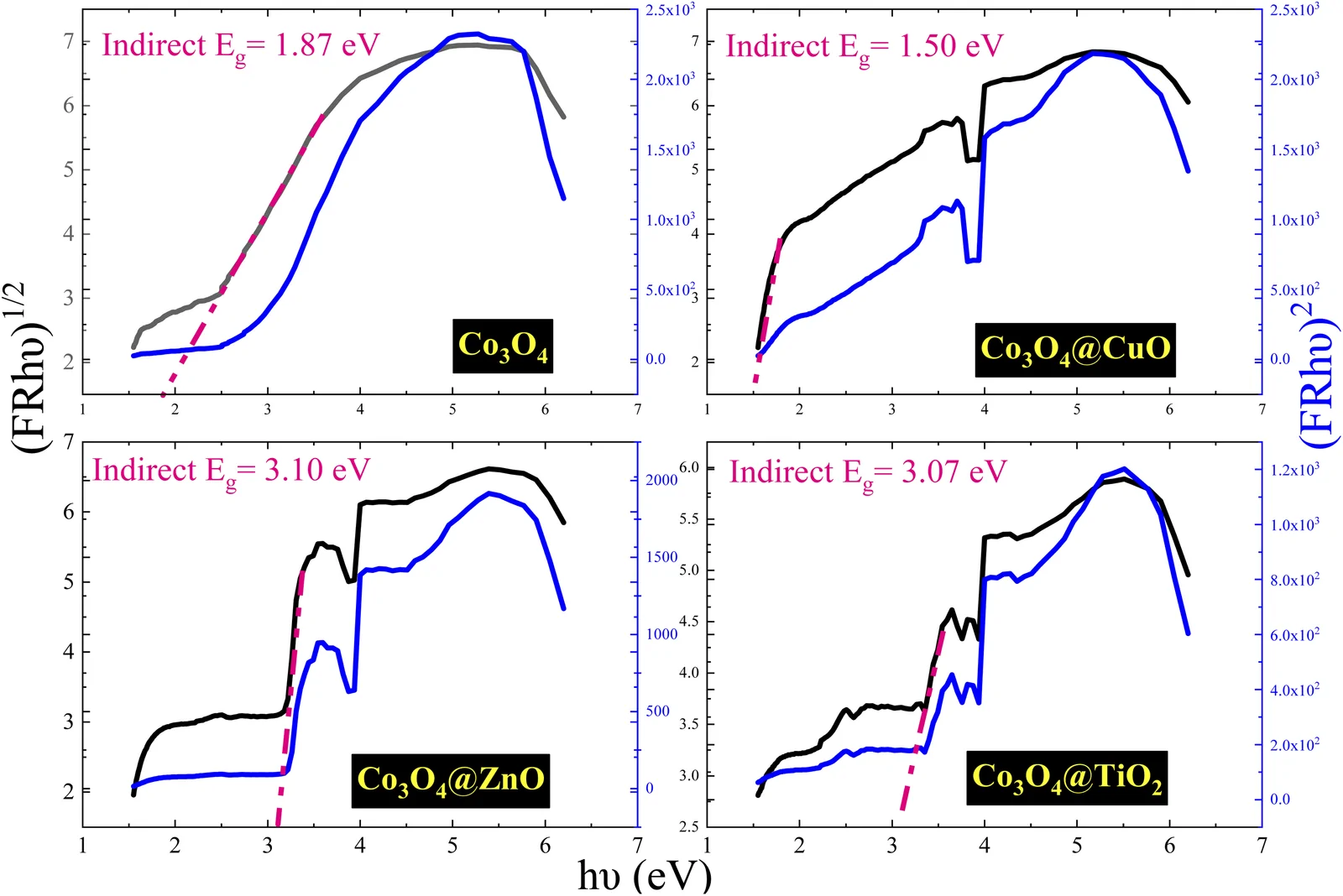
Tauc plots of Co_3_O_4_-M-O_*x*_ nanocomposite materials.

**Table 3:** Band gap energies of the synthesized materials with these of literature

	*E*_g_ (eV)	*E*_g_ literature values (eV)
CuO	—	1.2–1.8 (Ganga *et al.*)^69^
ZnO	—	3.15 (Dessie *et al.*)^67^
TiO_2_	—	3.66 (Zhang *et al.*)^68^
Co_3_O_4_	1.87	2.33 (Dessie *et al.*)^67^; 1.37 (Zhang *et al.*)^68^
Co_3_O_4_@ZnO	3.10	1.54 (Dessie *et al.*)^67^
Co_3_O_4_@CuO	1.50	—
Co_3_O_4_@TiO_2_	3.07	1.31 (Zhang *et al.*)^68^

DRS analysis revealed that the bandgap energy of the heterojunction was significantly lower than that of the pure oxides. The bandgap energies are therefore 3.10 eV for Co_3_O_4_@ZnO (compared to 3.15 eV for pure ZnO), 3.07 eV for Co_3_O_4_@TiO_2_ (compared to 3.66 eV for TiO_2_) and 1.50 eV for CoO_3_@CuO (compared to 1.8 eV for CuO). This reduction is attributed to orbital hybridisation (particularly between the cobalt 3d and oxygen 2p orbitals), interface defects, and band bending. This junction point is crucial for photon absorption and is located at the interface (Dessie and Zhang).^67,68^ Due to differences in the Fermi levels, structural defects and orbitals, as well as the curvature of the energy bands, contribute to reducing the width of the bandgap.

Consequently, as reported by Ganga and Santhosh in their study on CuO nanoparticle aggregation, electronic transitions occur at energies lower than the intrinsic band gaps of pure oxides. This process generates electron–hole pairs and increases the number of active sites per unit area, which could enhance the catalytic performance of these heterojunction materials.^69^

### Evaluation of photocatalytic efficiency

#### RhB and MB degradation profiles

This study evaluated the photocatalytic performance of cobalt(iii) oxide-based nanocomposite materials in degrading MB and RhB under UV and solar irradiation. Fig. 8 and 9 illustrate the changes in the absorption spectra of MB and RhB when exposed to UV light (efficient UV-induced activity is observed for the Co_3_O_4_@ZnO and Co_3_O_4_@TiO_2_ nanocomposites, and their MB and RhB degradation profiles are shown in Fig. S3 and S4). The results indicate that the degradation efficiency is approximately 90% for all nanocomposites within a timeframe of 120–135 minutes. Decreases in the maximum absorption bands, located at 650 nm for MB and 550 nm for RhB, demonstrate efficient dye degradation with increasing irradiation. This phenomenon is significantly more pronounced for the heterostructured materials than for pure Co_3_O_4_, as the interfaces between the Co_3_O_4_ and partner oxides appear to enhance the degradation process.^67–69^

**Fig. 8:**
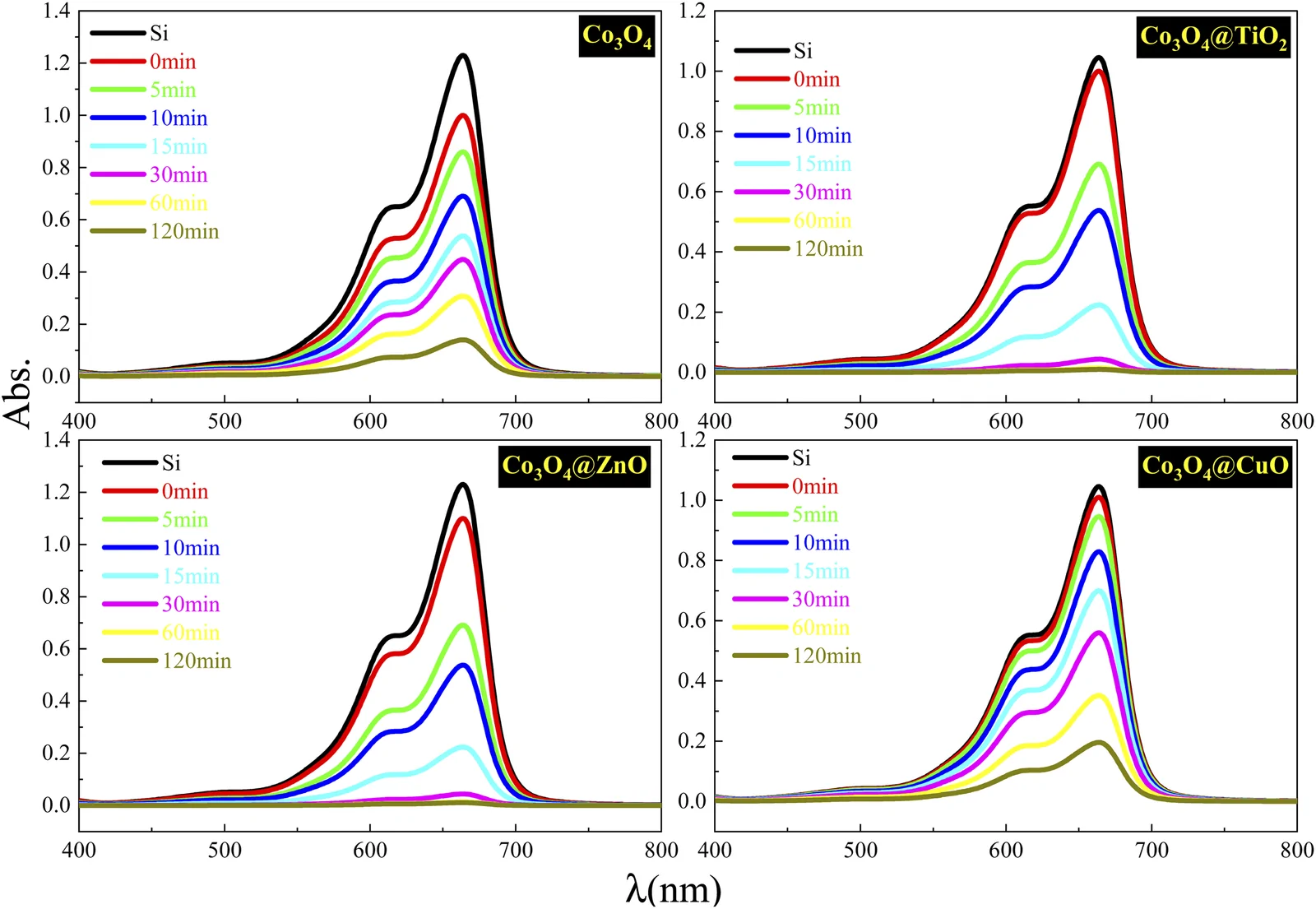
Evolution of MB solution absorption as a function of UV irradiation time in the presence of Co_3_O_4_-based composite catalysts.

**Fig. 9:**
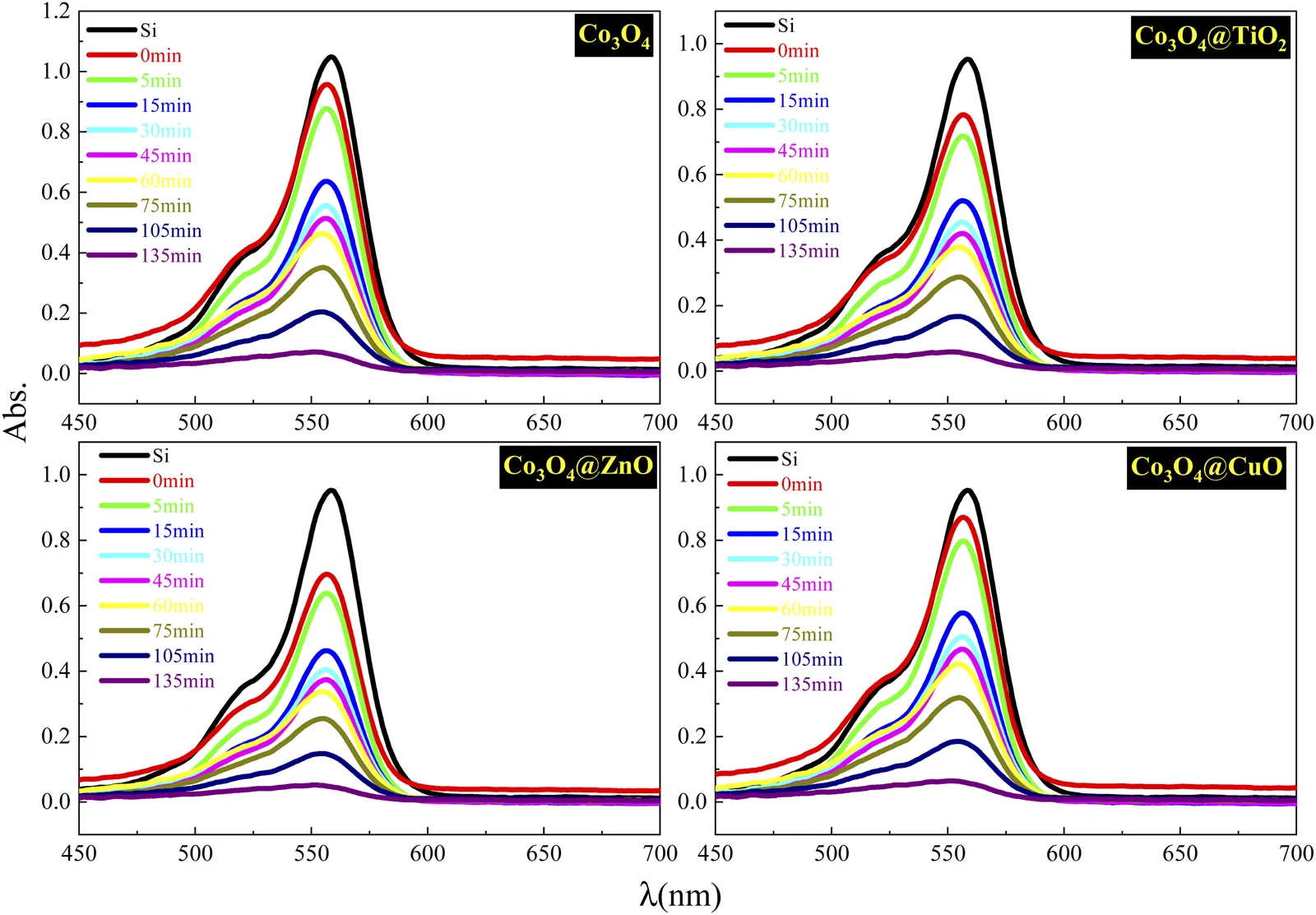
Evolution of RhB dye solution absorption as a function of UV irradiation time in the presence of Co_3_O_4_-based nanocomposite catalysts.

The proposed degradation mechanism is the same for all the synthesised nanocomposites and follows these steps:Co_3_O_4_@TiO_2_ + *hν* → Co_3_O_4_@TiO_2_(e_CB_^−^ + h_VB_^+^)e_CB_^−^ + O_2_ → ˙O_2_^−^h_VB_^+^ + H_2_O → ˙OH + H^+^h_VB_^+^ + OH^−^ → ˙OH•O_2_^−^ + H^+^ → ˙OOH2˙OOH → O_2_ + H_2_O_2_H_2_O_2_ + e_CB_^−^ → ˙OH + OH^−^

The superior photocatalytic performance of Co_3_O_4_@ZnO, Co_3_O_4_@TiO_2_ and Co_3_O_4_@CuO, compared to pure Co_3_O_4_, is primarily due to the formation of heterointerfaces between their constituent phases. In composite systems, the strategic integration of different metal oxides creates interfacial regions that act as active sites for charge transfer and surface reactions. These heterojunctions facilitate spatial charge separation, suppress bulk recombination, and enhance the overall quantum efficiency of photogenerated carriers. Unlike single-phase materials, where photogenerated electron–hole pairs rapidly recombine due to a narrow depletion layer and limited active surface sites, engineering nanocomposites promotes synergistic interfacial effects that improve photocatalytic functionality collectively (Kirty *et al.*).^70^

The enhanced activity observed for Co_3_O_4_@ZnO and Co_3_O_4_@TiO_2_ can be explained by the formation of p–n heterojunctions at the interface between the p-type Co_3_O_4_ and its respective n-type semiconductor oxide (either ZnO or TiO_2_). When they come into contact, the difference in their Fermi energy levels causes electrons to migrate from the n-type oxide (which has a higher Fermi level) to the p-type Co_3_O_4_ (which has a lower Fermi level), until thermal equilibrium is reached. This charge redistribution induces upward band bending in the n-type region and downward band bending in the p-type region, generating an internal electric field across the space-charge layer. Under illumination, this built-in potential acts as a driving force, directing photogenerated electrons towards the conduction band of the n-type oxide and holes towards the valence band of the p-type oxide. This vectorial charge transfer effectively mitigates electron–hole recombination, prolonging the lifetime of carriers and enabling separated charges to participate in interfacial redox reactions. Ultimately, this enhances photodegradation efficiency (Bharali *et al.*).^71^

In contrast, the Co_3_O_4_@CuO system forms a p–p heterojunction, where both components exhibit p-type semiconductor behavior. Consequently, the driving force for charge separation is weaker than in a p–n configuration, as the alignment of the two p-type materials' Fermi levels results in a less pronounced built-in potential and reduced band bending. Despite this, Co_3_O_4_@CuO exhibits superior photocatalytic performance under solar irradiation thanks to its exceptionally narrow bandgap of around 1.50 eV. This reduced optical band gap enables efficient harvesting of visible and near-infrared photons, which constitute a significant portion of the solar spectrum. The extended light absorption range compensates for the relatively diminished charge-separation efficiency by generating a higher population of photogenerated excitons, thereby offsetting the losses incurred from recombination at the p–p interface. This trade-off between light-harvesting capacity and charge-separation driving force emphasizes the need to balance optical and electronic properties when designing heterojunction photocatalysts.^71^

These findings are consistent with the broader nanocomposite engineering paradigm, which posits that combining materials with complementary electronic band structures, optical absorption characteristics, and morphological features can engender multifunctional properties surpassing those of the constituent parts. Similar synergistic effects have been widely reported in other multicomponent oxide systems, where phase coupling and interfacial charge redistribution can enhance the overall catalytic, electrochemical or photochemical performance beyond that of the pristine components (Abdullah *et al.*)^72^

Overall, the photocatalytic degradation profiles indicate that the formation of heterostructures is primarily responsible for the increased photoactivity of Co_3_O_4_-based nanocomposites. The exceptional performance of Co_3_O_4_@CuO under simulated solar light is primarily due to its extended visible-light absorption, which arises from the narrow bandgap of CuO. In contrast, the superior activity of Co_3_O_4_@ZnO and Co_3_O_4_@TiO_2_ is predominantly governed by efficient charge separation *via* the p–n heterojunction. Therefore, designing high-performance Co_3_O_4_-based photocatalysts requires balancing maximizing light absorption with optimizing interfacial charge dynamics. The choice of coupling oxide should be tailored to the specific illumination conditions and target application (Fig. 10).

**Fig. 10:**
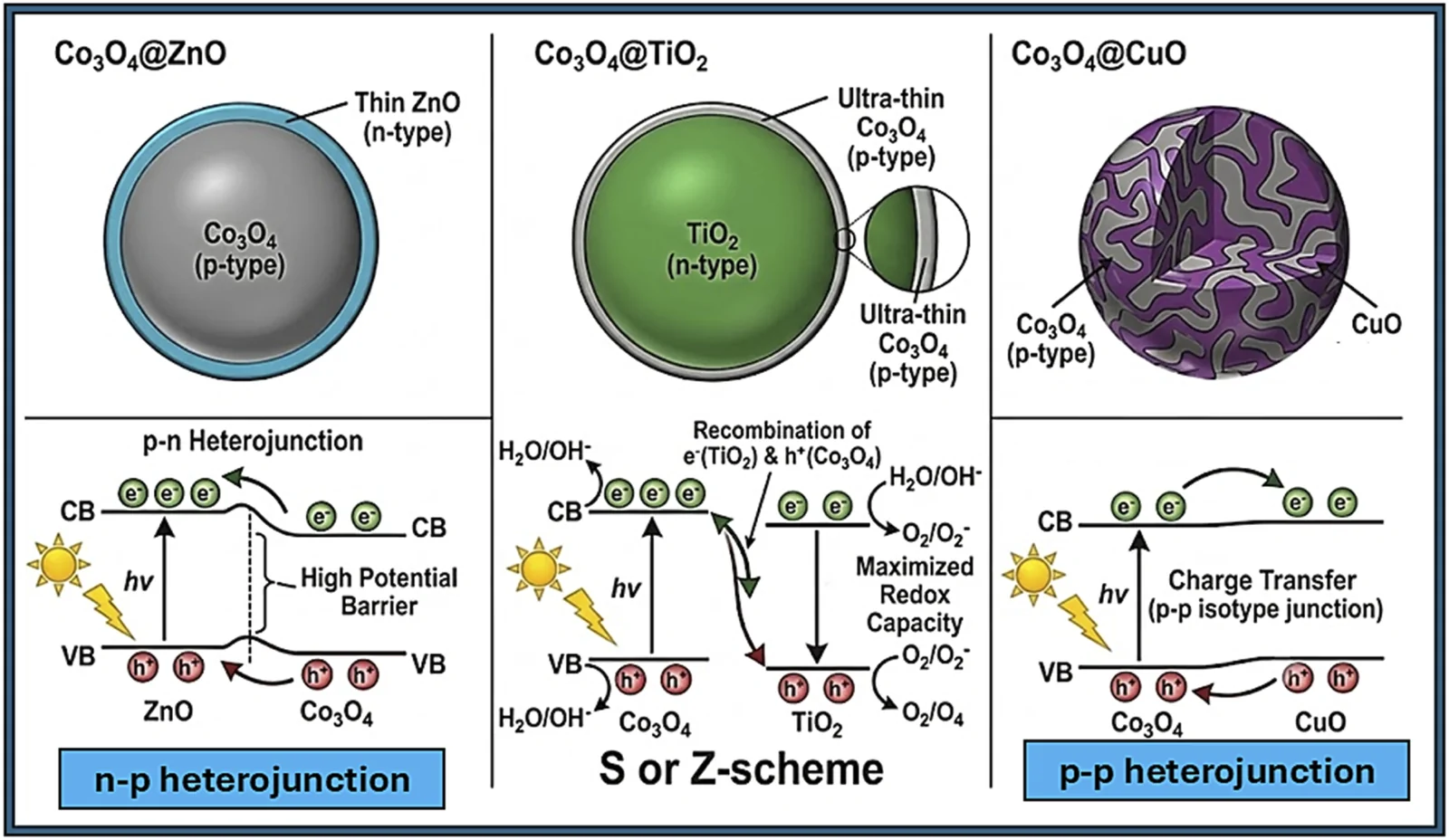
Proposed degradation mechanism of RhB and MB for the synthesized 700 nanocomposite materials.

#### Kinetics of RhB and MB degradation

Adjusting the experimental data to the pseudo-first-order kinetic model (Fig. 11) demonstrates excellent linearity (*R*^2^ > 0.99), confirming the model's suitability for describing the degradation of MB and RhB expressed as:^47^5
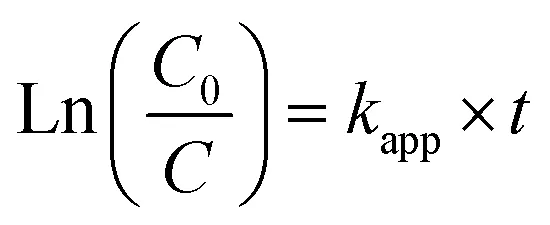


**Fig. 11:**
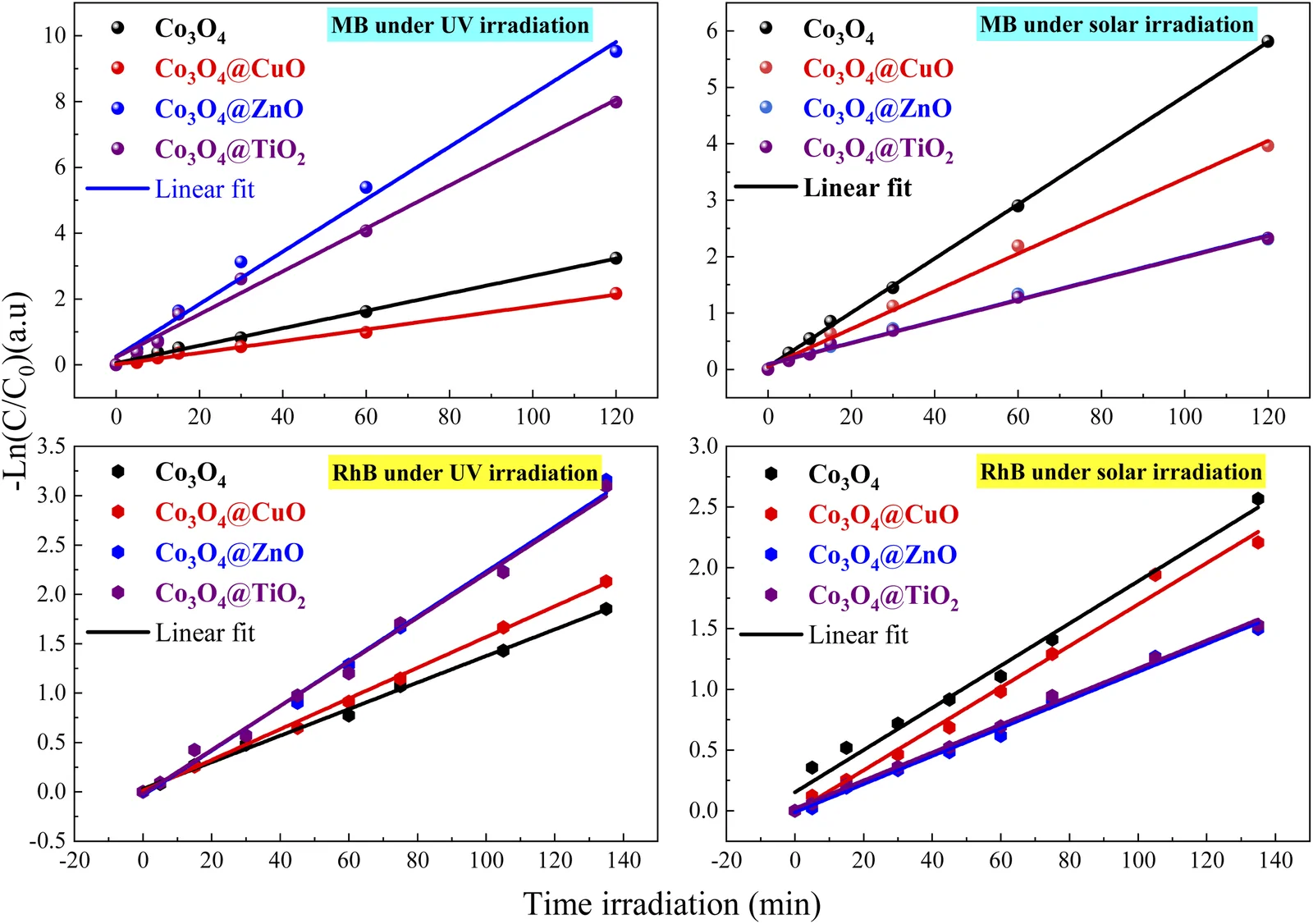
Kinetic pseudo-first-order profiles of MB and RhB under UV and solar light irradiations.

The model shows that the reaction rate is proportional to the colourant concentration. This indicates that the rate-limiting step is the surface reaction involving rapid adsorption (Rabani *et al.*).^73^

Table 4 shows the *K*_app_ values of the synthesised nanocomposites compared to values reported in the literature. The studied materials demonstrate superior degradation efficiency. Notably, the Co_3_O_4_@ZnO and Co_3_O_4_@TiO_2_ nanocomposites exhibited the fastest degradation rates and the highest Kappa values when exposed to UV light. This exceptional performance is attributed to the effective separation of photogenerated charges at the p–n heterojunction (Dessie *et al.*).^74^

**Table 4:** Summary of *k*_app_ values of different samples under UV and solar light with comparison with those in the literature

	Dye	Irradiation	*k*_app_ (min⁻¹)	Reference
Co_3_O_4_	MB	UV	∼0.026	Present study
Co_3_O_4_	RB	UV	∼0.013	Present study
Co_3_O_4_@CuO	MB	UV	∼0.017	Present study
Co_3_O_4_@CuO	RB	UV	∼0.015	Present study
Co_3_O_4_@ZnO	MB	UV	∼0.079	Present study
Co_3_O_4_@ZnO	RB	UV	∼0.023	Present study
Co_3_O_4_@TiO_2_	MB	UV	∼0.065	Present study
Co_3_O_4_@TiO_2_	RB	UV	∼0.022	Present study
ZnO/Co₃O₄	MB	UV	∼0.025	(Dessie *et al.*)^67^
Co₃O₄-TiO₂	MB	UV	∼0.030	(Zhang *et al.*)^68^
Co_3_O_4_	MB	Solar	∼0.048	Present study
Co_3_O_4_	RB	Solar	∼0.017	Present study
Co_3_O_4_@CuO	MB	Solar	∼0.033	Present study
CoO_3_@CuO	RB	Solar	∼0.017	Present study
Co_3_O_4_@ZnO	MB	Solar	∼0.019	Present study
Co_3_O_4_@ZnO	RB	Solar	∼0.011	Present study
Co_3_O_4_@TiO_2_	MB	Solar	∼0.019	Present study
Co_3_O_4_@TiO_2_	RB	Solar	∼0.011	Present study
Co_3_O_4_–CuO	MB	Solar	∼0.020	(Ganga *et al.*)^68^

The Co_3_O_4_@CuO nanocomposite exhibited the fastest degradation rate when exposed to sunlight, characterised by a significantly steeper kinetic slope. This is due to its narrow bandgap, which enhances light absorption within the visible spectrum.^69^

The degradation kinetics of RhB are generally slower than those of MB due to its more complex and stable xanthene ring structure. However, identical trends are observed across the materials, highlighting the beneficial role of heterojunction fabrication. Notably, a narrow bandgap alone—as observed in pristine Co_3_O_4_ (1.87 eV)—is insufficient to ensure favourable degradation kinetics.^69^

Consequently, the Co_3_O_4_@M-O_*x*_ (M = Zn, Cu, Ti; *n* = 1, 2) materials can be categorised as multicomponent semiconductor heterojunction nanocomposites. Their superior photocatalytic performance under UV and solar light indicates that the integrated heterostructure expands light-harvesting capabilities while optimising energy band alignment synergistically. This architectural configuration generates an internal electric field at the interface, promoting the rapid spatial separation and migration of photogenerated electron–hole pairs. Therefore, charge carrier recombination is significantly suppressed, resulting in an increased steady-state concentration of highly reactive oxygen species. The mineralisation kinetics of RhB and MB are accelerated by the abundance of these species, ultimately resulting in the fastest observed reaction rate constants, as demonstrated by several studies (Souamti, Alharthi, Zhang).^75–78^

### Electrical properties

#### Nyquist diagrams

Nyquist plots of synthesised nanocomposite materials reveal distinct semicircular arcs that change significantly with temperature. Specifically, the diameter of the semicircles decreases systematically as the temperature rises from 100 °C to 200 °C (Fig. 12). This behaviour is indicative of a semiconductor with a negative temperature coefficient of resistance (NTCR). This phenomenon is attributed to thermally activated charge carrier hopping, which reduces the nanocomposites' overall impedance at elevated temperatures (Souemti *et al.*).^79^ Of the materials tested, pure Co_3_O_4_ exhibited the highest level of resistance (1.5 × 10^4^ Ω), displaying a broad, dominant semicircle primarily associated with grain (or bulk) resistance. In contrast, the heterojunction with metallic oxides significantly reduced the resistance scale to 10^1^ and 10^2^ Ω (Table 5) and produced ‘depressed’ semicircles. The centres of these arcs are shifted below the real axis. This phenomenon is typically caused by structural heterogeneity, including surface roughness, varying porosity, and the distribution of grain boundaries.^79^ The high-frequency arcs indicate conduction within the grains. However, the secondary responses observed at lower frequencies suggest that grain boundaries and the interfaces between the Co_3_O_4_ matrix and metal oxides play a significant role in conduction.^79^

**Fig. 12:**
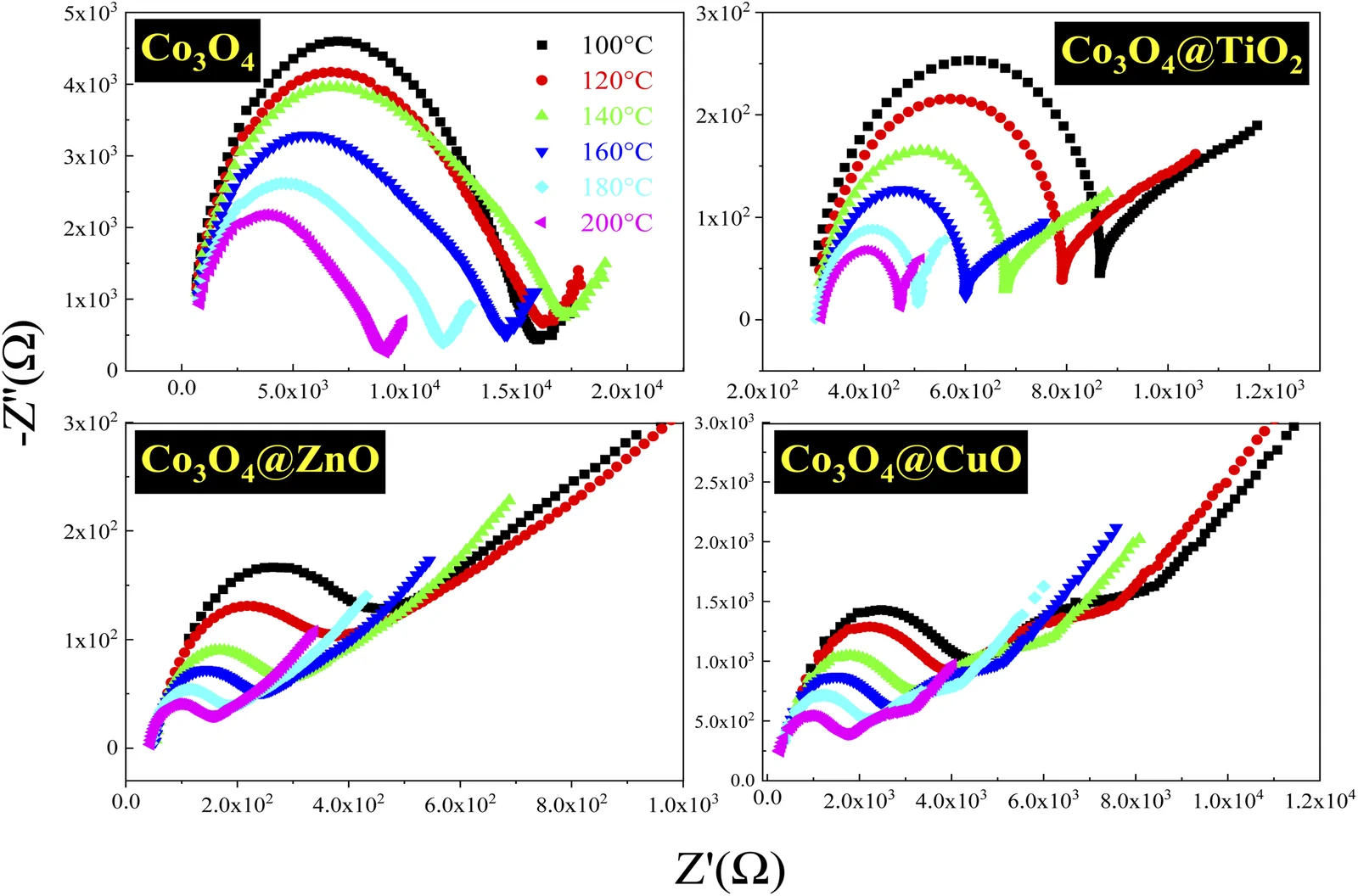
Nyquist diagrams of the synthesized nanocomposite materials.

**Table 5:** Resistance and conductivity values as a function of temperature for the Co_3_O_4_-based nanocomposite materials

*T* (°C)	Co_3_O_4_		Co_3_O_4_@TiO_2_		Co_3_O_4_@ZnO		Co_3_O_4_@Cuo	
	*R* (10^−6^ Ω)	*σ* (10^6^ S cm^−1^)	*R* (10^2^ Ω)	*σ* (10^−4^ S cm^−1^)	*R* (10^2^ Ω)	*σ* (10^6^ S cm^−1^)	*R* (10^3^ Ω)	*σ* (10^5^ S cm^−1^)
100	1.472	4.22	2.922	2.13	2.713	2.29	3.022	2.06
120	1.269	4.89	2.482	2.50	2.331	2.66	2.721	2.28
140	1.191	5.21	1.899	3.27	1.5 56	3.99	2.103	2.96
160	1.084	5.72	1.532	4.05	1.216	5.10	1.900	3.28
180	1.026	6.05	1.069	5.81	0.983	6.95	1.554	4.01
200	0.991	6.26	0.801	7.75	0.734	8.45	1.156	5.41

These responses were particularly pronounced in the CuO and ZnO heterojunction nanocomposites. Therefore, it appears that the nanocomposite structure facilitates enhanced charge transport pathways compared to the pure oxide, as demonstrated by the transition from a single arc to more complex, overlapping features.

As the Nyquist plots show, the Co_3_O_4_ nanocomposite exhibited p-type semiconducting behaviour. This can be explained by thermal activation facilitating a small polaron hopping mechanism between Co^2+^ and Co^3+^ ions. TiO_2_, ZnO or CuO heterojunctions with Co_3_O_4_, which have high bulk resistance, substantially reduce impedance by forming p–n or p–p heterojunctions. This enhances charge transport across interfaces (Lu, Xu).^80,81^

The ‘depressed’ semicircles therefore indicate the presence of a constant phase element (CPE), which results from structural heterogeneity including surface porosity and grain boundary distribution. Consequently, the transition from high-frequency bulk arcs to complex low-frequency features suggests that the conduction mechanism is primarily influenced by the interaction between the grain interiors and the interfacial barriers of the nanocomposite matrix.^75^ Nyquist analysis therefore confirmed that the heterojunction significantly improved the electrical conductivity of cobalt oxide, classifying these materials as low-temperature, functionalised semiconductor nanocomposites that conduct electrons and holes.

Consequently, these Co_3_O_4_-based heterojunction nanocomposites are most used as supercapacitor electrodes in energy devices due to their interfacial synergy and improved conductivity, which facilitates faster ion and electron transport.^32^ These nanocomposites have been shown to consistently increase capacitance and stability across multiple systems. The same conductivity gains can be exploited to create electrodes that facilitate water splitting and hydrogen production.^60^ Furthermore, these nanocomposites are equally effective in battery-type systems, including zinc-air batteries and flexible solid-state storage devices. In these systems, enhanced interfacial charge transfer improves power output, cycling durability, and the practical operation of devices.^81,82^

#### Arrhenius plot analysis

Fig. 13 illustrates the dependence of the conductivity of nanocomposite materials on temperature. Conductivity is thermally activated. The activation energies *E*_a_ were calculated by fitting the conductivity data to the Arrhenius equation for thermally activated conduction.:^83^6
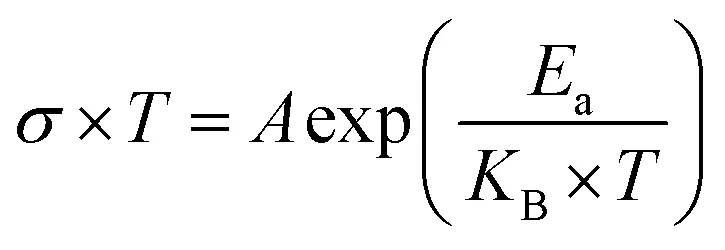
where *T* is the absolute temperature (K), *K*_B_ is the Boltzmann constant, and *A* is the preexponential factor.

**Fig. 13:**
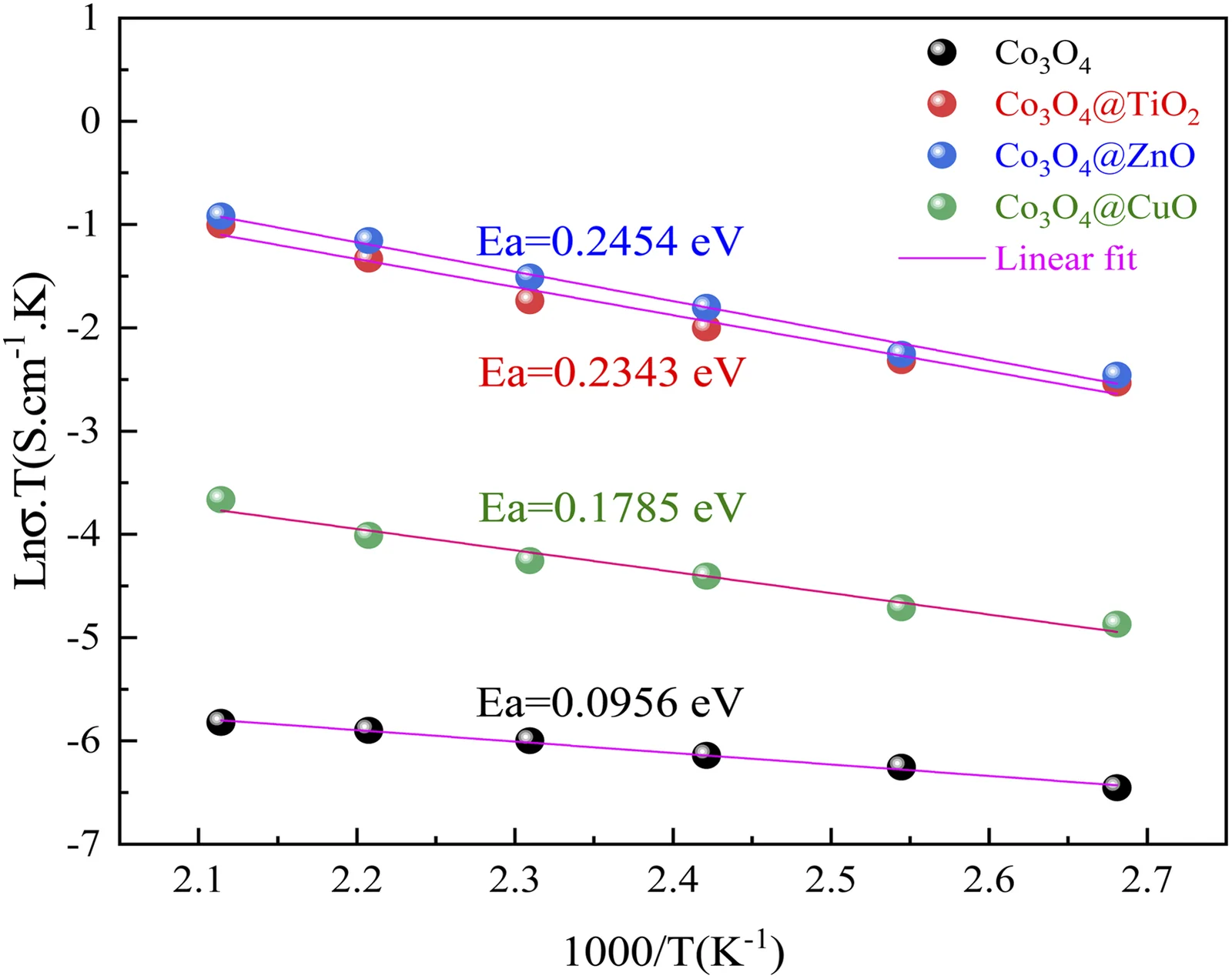
Arrhenius plots and activation energies of Co_3_O_4_-based nanocomposites.

The activation energy values have been shown to quantify the energy barrier for small polaron hopping between mixed-valence metal ions.

Notably, the activation energy of Co_3_O_4_ is exceptionally low at 0.0956 eV. This can be explained by an efficient intrinsic hopping process within the pure spinel lattice. However, the increased values observed for the nanocomposites suggest the presence of interfacial energy barriers within the heterojunctions.

The elevated activation energies of 0.2454 eV for Co_3_O_4_@ZnO and 0.2343 eV for Co_3_O_4_@TiO_2_ are indicative of p–n heterojunctions, where significant potential barriers for hole transport are created by the formation of depletion regions at the grain boundaries.^48^ However, with a value of 0.1785 eV, Co_3_O_4_@CuO has a lower energy barrier than the other composite materials due to favourable band alignment at the p–p isotype junction. This facilitates enhanced charge transfer across the interface. In summary, the obtained values fall within the range of 0.1–0.4 eV, confirming that electronic hopping at the grain boundaries is the predominant conduction mechanism.

#### Jonsher's power law analysis

Fig. 14a illustrates the variation of AC conductivity (*σ*_ac_) with frequency at various temperatures. Jonscher's power law is a well-known method of analysing conductivity dispersion in solids. The law is specified as follows:^84^

7 *σ*_ac_(*ω*) = *σ*_dc_ + *Aω*^*S*^

where *A* denotes a temperature-dependent parameter; ‘*s*’ is the temperature-dependent exponent in the 0 ≤ *s* ≤ 1 range; and *σ*_dc_ is the dc conductivity, which is calculated by extrapolating the curves of *σ*_ac_ to zero frequency at varying temperatures. The separation between the two frequency regions occurs at a transition point in the slope that corresponds to a given temperature. This frequency is known as the ‘hopping frequency’, or *ω*_p_. It has been observed that the values of *ω*_p_ increase proportionally with temperature.^73^

**Fig. 14:**
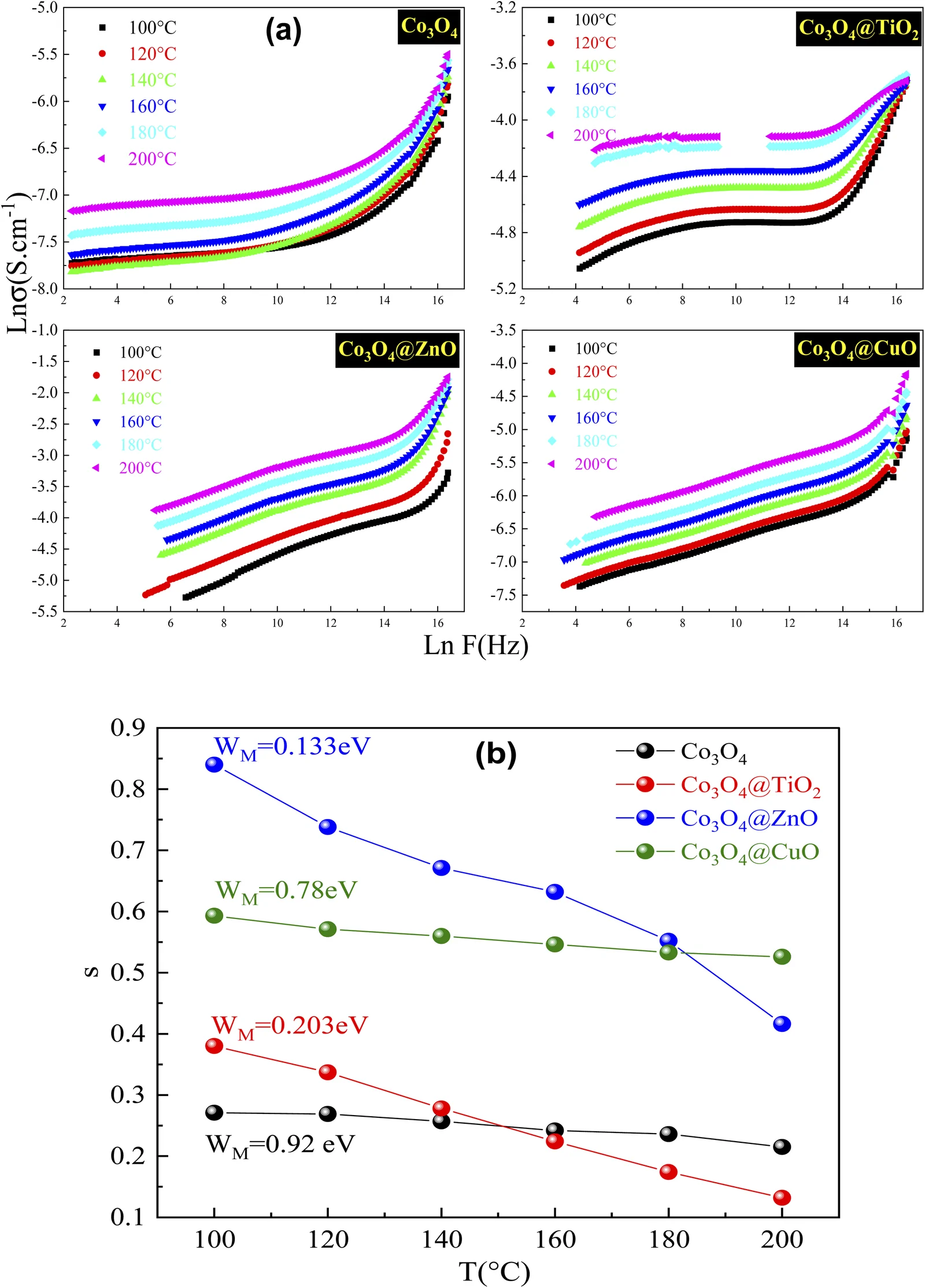
Variation of conductivities as a function of frequency at different temperatures (a), Jonsher's power law showing the ‘*s*’ and WM values at higher frequencies (b).

Additionally, the conductivity demonstrated linear behavior at higher frequencies for all the samples. In such cases, conductivity depends on frequency according to the equation:

8 *σ*_ac_ ≈ *A*·*ω*^*s*^

A thermally activated process indicating hopping conduction between states at higher frequencies is responsible for this behavior.^73^

As demonstrated in Fig. 14(b), the ‘*s*’ values are derived from the slopes of the *σ*_ac_ = *f*[ln(*f*)] plots at higher frequencies. The ‘*s*’ is independent of temperature. However, as the temperature increases, this value decreases. This process is known as correlated barrier hopping (CBH) and is defined as the simultaneous movement of two charge carriers over a potential barrier that separates two defect sites.^73^

Once the value of *s* has been obtained, the quantity of energy necessary for the removal and subsequent transfer of a charge carrier from one site to another *W*_M_ (eV) can be calculated *via* the following formula:^79^9
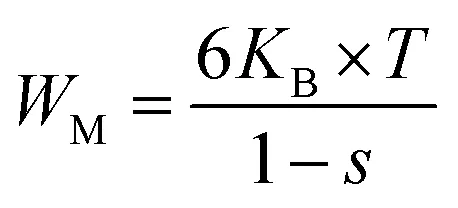


For pure Co_3_O_4_, the values of *s* are observed to vary between 0.2 and 0.3, with an estimated barrier energy (WM) of approximately 0.92 eV. This behaviour indicates a relatively difficult charge-hopping process due to strong repulsion between localised charge carriers (Gupta).^84^ In contrast, the values of *s* for Co_3_O_4_@TiO_2_ (0.1–0.4) and Co_3_O_4_@ZnO (0.4–0.9) correspond to significantly lower barrier heights of 0.203 and 0.133 eV, respectively. This is due to the heterojunctions, which introduce intermediate defect states and thus facilitate charge transfer. In this case, the p–n junction has a wide range of barrier heights and a temperature-dependent hopping mechanism which becomes increasingly delocalised at high temperatures (Szatan, Moualhi).^85,86^

Conversely, the Co_3_O_4_@CuO material has a high barrier height of 0.78 eV (with *s* = 0.5–0.6), indicating that this nanocomposite is less effective at reducing the jump barrier due to weaker interfacial coupling (Moualhi).^87^

In conclusion, materials governed by the CBH phenomenon or mixed hopping are often recommended for energy storage applications. Their transport properties can be tailored according to composition, defects, and interfaces, making them promising candidates for use as sensors in nanoscale semiconductor devices and electrochemical systems (Biswas, Yassine).^88,89^

### Correlation of textural, catalytic and electrical properties

The experimental results clearly demonstrate a consistent relationship between the textural structure, catalytic performance, and electrical properties of the materials, which is due to the heterojunction process involving various metal oxides. Firstly, an inverse correlation was observed between the specific surface area and the bandgap energy for n-type heterojunctions. In this case, the Co_3_O_4_@TiO_2_ material exhibits the highest *S*_BET_ (5.116 m^2^ g^−1^) and the widest bandgap (3.07 eV), whereas the Co_3_O_4_@ZnO material has a moderate SBET (2.985 m^2^ g^−1^) and a wider bandgap (3.10 eV). In contrast, pure spinel Co_3_O_4_ and the p-type heterojunction (Co_3_O_4_@CuO) have lower *S*_BET_s (3.488 and 3.684 m^2^ g^−1^ respectively) and narrower band gaps (1.87 and 1.50 eV respectively). This suggests that a higher SBET does not necessarily correspond to a narrower bandgap for n-type heterojunctions. In fact, the electronic structure is more strongly influenced by interfacial charge transfer and the intrinsic properties of the secondary semiconductor. Nevertheless, the higher *S*_BET_ of the Co_3_O_4_@TiO_2_ material provides a significant textural advantage, offering more active sites for dye adsorption—a prerequisite for efficient photocatalysis.

When exposed to UV light, p–n heterojunctions (Co_3_O_4_@ZnO and Co_3_O_4_@TiO_2_) show significantly higher degradation rates for RhB and MB than pure Co_3_O_4_ or p–p heterojunctions (Co_3_O_4_@CuO). This is attributed to the formation of an internal electric field at the p–n interface, which separates photogenerated electron–hole pairs effectively and reduces recombination. Furthermore, the Co_3_O_4_@TiO_2_ material has a wider bandgap (3.07 eV) than the Co_3_O_4_@CuO material (1.50 eV). This effective charge separation makes the Co_3_O_4_@TiO_2_ material more active under UV light, compensating for its lower absorption in the visible spectrum. In contrast, the p–p heterojunction (Co_3_O_4_@CuO) exhibits lower UV activity due to its p–p type, resulting in less efficient band bending and charge separation. However, under solar irradiation, which contains a significant visible light component, the p–p heterojunction outperforms the p–n types. This reversal can be explained by the narrower bandgap of CuO (1.50 eV), which enables broader absorption of the solar spectrum and generates more charge carriers, despite less efficient separation.

Measuring electrical conductivity as a function of frequency within the temperature range of 100 to 200 °C provides further insight into the heterojunction effect. The formation of heterojunctions has been shown to consistently improve electrical conductivity compared to pure Co_3_O_4_ in the following order: Co_3_O_4_ < Co_3_O_4_@ZnO < Co_3_O_4_@CuO < Co_3_O_4_@TiO_2_. This trend correlates remarkably well with photocatalytic activity under solar irradiation. Co_3_O_4_@TiO_2_ and Co_3_O_4_@CuO demonstrate optimal performance in sunlight due to their high conductivity. This improvement in conductivity is a direct consequence of the heterojunction interface, which facilitates charge carrier transport and reduces volume resistance. However, the order does not strictly follow the specific surface area values, suggesting that electrical transport is more sensitive to the alignment of electronic bands and interface quality than to SBET alone. For instance, Co_3_O_4_@TiO_2_, which has the highest SBET and the widest bandgap, exhibits the best conductivity. This may be because its large *S*_BET_ improves intergranular conduction, or it may be that the TiO_2_ interface provides a more efficient pathway for charge transfer.

This electrical behavior demonstrates that heterojunctions enhance charge separation and macroscopic transport properties. The latter is crucial for driving surface redox reactions in photocatalysis.

In summary, the results reveal multidimensional interactions: textural properties facilitate adsorption, while the electronic structure—in terms of the bandgap and type of heterojunction—controls charge separation and light absorption. Electrical conductivity, meanwhile, reflects the efficiency of overall charge transport. p–n heterojunctions perform better under UV light due to efficient charge separation. Co_3_O_4_@TiO_2_ additionally benefits from a higher specific surface area. Conversely, the p–p heterojunction excels under solar irradiation thanks to its narrow bandgap and improved conductivity, despite its lower specific surface area. Therefore, the heterojunction effect consistently enhances both electrical conductivity and catalytic activity compared to pure Co_3_O_4_, with optimal performance depending on the light source.

These results emphasize that, for solar applications, achieving a balance between a narrow bandgap for light absorption and charge separation induced by the heterojunction is more important than maximizing the specific surface area. However, a p–n heterojunction with a higher specific surface area is preferable for UV processes.

## Conclusions

In this study, we successfully synthesised Co_3_O_4_@M-O_*x*_ (where M = Zn, Ti or Cu) heterojunctions *via* a simple hydrothermal process. This yielded pure crystalline phases with nanoscale particle sizes of ≤98 nm, as well as cubic spinel Co_3_O_4_ fractions ranging from 21.38% to 64.21%. The resulting p–n, p–p and Z-scheme architectures gave the ZnO (3.10 eV) and TiO_2_ (3.07 eV) composites wide band gaps. These architectures enabled 90% photocatalytic degradation of RhB and MB within 135 minutes under pseudo-first-order kinetics, achieving significantly better performance than pristine Co_3_O_4_.

These materials act as dual-type semiconductors at low temperatures (100–200 °C), with transport being governed either by CBH or by mixed hopping. Compared to pure spinel, heterojunction effects markedly enhance conductivity.

The results reveal multidimensional interactions: textural properties facilitate adsorption, while the electronic structure controls charge separation and light absorption in terms of the bandgap and heterojunction type. Meanwhile, electrical conductivity reflects the efficiency of overall charge transport. p–n heterojunctions perform better under UV light due to their ability to separate charges efficiently.

Interfacial synergy enhances the catalytic and electrochemical performance of these nanocomposites, making them promising candidates for use in supercapacitors, sensors and electrochemical systems.

Future research will focus on integrating biomaterials or constructing ternary Co_3_O_4_-based heterostructures to promote sustainable hydrogen production within the context of a green economy. Overall, this work provides a scalable, dual-purpose platform for environmental remediation and next-generation energy storage.

## Author contributions

Ahmed Souamti: conceptualization, investigation, funding acquisition, writing – original draft, writing – review & editing, visualization, validation, methodology, software, formal analysis, data curation. Radhouene Kahlaoui: conceptualization, investigation, funding acquisition, visualization, validation, methodology, data curation, software, writing – review & editing, writing – original draft. Abderraouf Jraba: investigation, writing – original draft, funding acquisition, visualization, writing – review & editing, formal analysis, data curation. Muhammad Humayun: resources, data curation, visualization, writing – review & editing, funding acquisition, methodology, conceptualization, software, formal analysis. Mohamed Bououdina: validation, resources, visualization, writing – review & editing conceptualization, acquisition. Adel Megriche: resources, supervision, data curation, project administration, visualization, writing – review & editing, funding acquisition, methodology, conceptualization. Latifa Latrous: project administration, resources, supervision, visualization, writing – review & editing, conceptualization.

## Conflicts of interest

The authors declare no conflicts of interests in this work.

## Supplementary Material

RA-016-D6RA06628C-s001

## Data Availability

The datasets generated and/or analysed during the current study are available from the corresponding authors upon reasonable request. Supplementary information (SI) is available. See DOI: https://doi.org/10.1039/d6ra06628c.

## References

[cit1] You W., Yun T., Liu J. (2026). *et al.*, Breaking the toughness–strength trade-off in polymer nanocomposites via a mechanically interlocked interface. Nat. Commun..

[cit2] Melegy A. A., Zaki E. G., El-Saeed S. M. (2026). *et al.*, Removal of toxic hexavalent chromium ions from water using magnetic protic poly (ionic liquids) nanocomposites. Sci. Rep..

[cit3] Thiru S., Salem Said A. H. S., Megahed M. (2026). *et al.*, Advanced hybrid nanocomposites for infrastructure renewal: enhanced shear and flexural performance in load-bearing applications. Sci. Rep..

[cit4] Fahidazar M. A., Afghahi S. S. S., Beyranvand M. (2026). MXene@Cu@PPy nanocomposites with enhanced electromagnetic response. Sci. Rep..

[cit5] Haghir Madadi M., Ehsani M., Naderi G. (2026). Tailoring the performance of polypropylene nanocomposites with hybrid fillers for high-voltage electrical applications. Sci. Rep..

[cit6] Dhakshinamoorthy A., Li Z., Yang S., García H. (2024). Metal-organic framework heterojunctions for photocatalysis. Chem. Soc. Rev..

[cit7] Kosco J., González-Carrero S., Howells C. T. (2022). *et al.*, Generation of long-lived charges in organic semiconductor heterojunction nanoparticles for efficient photocatalytic hydrogen evolution. Nat. Energy.

[cit8] Joseph S., Mohan J., Lakshmy S. (2023). *et al.*, A review of the synthesis, properties, and applications of 2D transition metal dichalcogenides and their heterostructures. Mater. Chem. Phys..

[cit9] Zhu Y. (2025). Creating multifunctionality with hierarchical heterostructures. Mater. Res. Lett..

[cit10] Nepal D., Kang S., Adstedt K. M. (2022). *et al.*, Hierarchically structured bioinspired nanocomposites. Nat. Mater..

[cit11] Yuan S., Duan X., Liu J., Ye Y. (2021). *et al.*, Recent progress on transition metal oxides as advanced materials for energy conversion and storage. Energy Storage Mater..

[cit12] Flores-Lasluisa J., Huerta F., Cazorla-Amorós D., Morallón E. (2022). Transition metal oxides with perovskite and spinel structures for electrochemical energy production applications. Environ. Res..

[cit13] Tatrari G., Ahmed M., Shah F. U. (2024). Synthesis, thermoelectric and energy storage performance of transition metal oxides composites. Coord. Chem. Rev..

[cit14] Kumar S., Tahira A., Bhatti A. L. (2024). *et al.*, Oxygen vacancies and tailored redox activity encountered with NiCo_2_O_4_ nanostructures for promising applications in supercapacitor and water oxidation. J. Energy Storage.

[cit15] Yin M., Miao H., Hu R., Sun Z., Li H. (2021). Manganese dioxides for oxygen electrocatalysis in energy conversion and storage systems over full pH range. J. Power Sources.

[cit16] Lei Z., Lee J. M., Singh G. (2021). *et al.*, Recent advances of layered-transition metal oxides for energy-related applications. Energy Storage Mater..

[cit17] Vazhayil A., Vazhayal L., Thomas J. (2021). *et al.*, A comprehensive review on the recent developments in transition metal-based electrocatalysts for oxygen evolution reaction. Appl. Surf. Sci. Adv..

[cit18] Reddy N. R., Kumar A. S., Reddy P. M. (2023). *et al.*, Novel rhombus Co_3_O_4_-nanocapsule CuO heterohybrids for efficient photocatalytic water splitting and electrochemical energy storage applications. J. Environ. Manage..

[cit19] Du C., Zhao Z., Liu H. (2025). *et al.*, Novel ZnO/NiO heterostructures with defects: An outstanding electrode material for high-performance supercapacitors. J. Energy Storage.

[cit20] Zhang Y., Liao W.-M., Zhang G. (2021). A general strategy for constructing transition metal oxide/CeO_2_ heterostructure with oxygen vacancies toward hydrogen evolution reaction and oxygen evolution reaction. J. Power Sources.

[cit21] Balasubramanian V., Shunmugapriya B., Suman R. (2025). *et al.*, Bifunctional heterostructure ZnWO_4_@ZnO nanocomposite for high-performance electrocatalysis and supercapacitor applications. Mater. Sci. Eng., B.

[cit22] Ramgopal N., Roy N., Sreedevi G. (2025). *et al.*, Construction of a hybrid electrocatalyst by impregnating the CuS/CdFe_2_O_4_ composite heterostructure into the carbon nanofiber network for improved electrocatalytic oxygen evolution reaction. J. Mol. Liq..

[cit23] Vattikuti S. V. P., Goud J. P., Aljuwayid A. M. (2024). *et al.*, Zr/Ni metal oxide nanostructures: electrochemical explore and urea oxidation catalysts. Ceram. Int..

[cit24] Li B., Ji D., Hamouda A. K., Luo S. (2024). MXene-derived TiO_2_ nanosheets/rGO heterostructures for superior sodium-ion storage. ChemPhysMater.

[cit25] Sahu D., Panda N. R. (2023). Synthesis of novel nanocomposite of g-C_3_N_4_ coated ZnO-MoS_2_ heterostructure for energy storage and photocatalytic applications. Chemosphere.

[cit26] Bai L., Huang H., Yu S. (2021). *et al.*, Role of transition metal oxides in g-C_3_N_4_-based heterojunctions for photocatalysis and supercapacitors. J. Energy Chem..

[cit27] Ali S. A., Ahmad T. (2023). Enhanced hydrogen generation via overall water splitting using novel MoS_2_-BN nanoflowers assembled TiO_2_ ternary heterostructures. Int. J. Hydrogen Energy.

[cit28] Ahmed M. S., Choi B., Kim Y. (2018). Development of highly active bifunctional electrocatalyst using Co_3_O_4_ on carbon nanotubes for oxygen reduction and oxygen evolution. Sci. Rep..

[cit29] Chauhan A., Kumar R., Devi S. (2024). *et al.*, Recent advances on Co_3_O_4_-based nanostructure photocatalysis: Structure, synthesis, modification strategies, and applications. Surf. Interfaces.

[cit30] Zhao F., Ma H. (2023). Application of Co_3_O_4_ in photoelectrocatalytic treatment of wastewater polluted with organic compounds: A review. Crystals.

[cit31] Nashim A., Pany S., Parida K. (2024). Effect of synthesis methods on the activity of NiO/Co_3_O_4_ as an electrode material for supercapacitor: In the light of X-ray diffraction study. RSC Adv..

[cit32] Sivaramalakshmi S., Murugan D., Kumar M. R. (2025). *et al.*, Synthesis of heterostructure CeO_2_–Co_3_O_4_ nanocomposite by hydrothermal approach for high performance supercapacitor application. Mater. Today Commun..

[cit33] Park J.-W., Ju Y.-W. (2023). Evaluation of bifunctional electrochemical catalytic activity of Co_3_O_4_–CoFe_2_O_4_ composite spinel oxide. Energies.

[cit34] Zia A., Naveed A. B., Javaid A., Ehsan M. F., Mahmood A. (2022). Facile synthesis of ZnSe/Co_3_O_4_ heterostructure nanocomposites for the photocatalytic degradation of Congo Red dye. Catalysts.

[cit35] Naikoo G. A., Tabook M. A., Tabook B. (2024). *et al.*, Electrochemical performance of Co_3_O_4_/Ag/CuO electrodes for supercapacitor applications. J. Energy Storage.

[cit36] Bathula C., Rabani I., Ramesh S. (2021). *et al.*, Highly efficient solid-state synthesis of Co_3_O_4_ on multiwalled carbon nanotubes for supercapacitors. J. Alloys Compd..

[cit37] Wang Z.-Y., Jiang S., Duan C. (2020). *et al.*, In situ synthesis of Co_3_O_4_ nanoparticles confined in 3D nitrogen-doped porous carbon as an efficient bifunctional oxygen electrocatalyst. Rare Met..

[cit38] Araei Z., Ghorbani M. (2025). Synergistic effect of Co_3_O_4_/CdAl_2_O_4_ nanocomposite for photocatalytic decontamination of organic dyes under visible light irradiation. Sci. Rep..

[cit39] Malefane M. E. (2020). Co_3_O_4_/Bi_4_O_5_I_2_/Bi_5_O_7_I C-scheme heterojunction for degradation of organic pollutants by light-emitting diode irradiation. ACS Omega.

[cit40] Yousefi S. R., Alshamsi H. A., Amiri O., Salavati-Niasari M. (2021). Synthesis, characterisation and application of Co/Co_3_O_4_ nanocomposites as an effective photocatalyst for discolouration of organic dye contaminants in wastewater and antibacterial properties. J. Mol. Liq..

[cit41] Xiao J., Kuang Q., Yang S. (2013). *et al.*, Surface structure dependent electrocatalytic activity of Co_3_O_4_ anchored on graphene sheets toward oxygen reduction reaction. Sci. Rep..

[cit42] Han X., He G., He Y. (2018). *et al.*, Engineering catalytic active sites on cobalt oxide surface for enhanced oxygen electrocatalysis. Adv. Energy Mater..

[cit43] Babu C., Avani A., Xavier T. (2024). *et al.*, Symmetric supercapacitor based on Co_3_O_4_ nanoparticles with an improved specific capacitance and energy density. J. Energy Storage.

[cit44] Karthikeyan A., D R., Bakkiyaraj A. R., Krishnamoorthy E. (2023). High electrochemical performance of Co_3_O_4_-PVDF-NMP-based supercapacitor electrode. J. Mater. Sci.: Mater. Electron..

[cit45] Shah M. S., Iqbal Z., Ul-Hamid A., Mushtaq M. U., Azad F. (2025). Tailoring the electrochemical performance of rods-like Co-MOF:Fe-derived Co_3_O_4_:Fe electrodes for supercapacitor applications. Fuel.

[cit46] Dhakal G., Mohapatra D., Tamang T. (2021). *et al.*, Redox-additive electrolyte–driven enhancement of the electrochemical energy storage performance of asymmetric Co_3_O_4_//carbon nano-onions supercapacitors. Energy.

[cit47] Souemti A., Jraba A., Kahlaoui R., Elaloui E., Latrous L. (2025). Beyond conventional MoO_3_: Integrated design of composite materials for dual catalytic and energy storage applications. Ceram. Int..

[cit48] Shi Z., Lan L., Li Y. (2018). *et al.*, Co_3_O_4_/TiO_2_ nanocomposite formation leads to improvement in ultraviolet–visible-infrared-driven thermocatalytic activity due to photoactivation and photocatalysis–thermocatalysis synergetic effect. ACS Sustainable Chem. Eng..

[cit49] Han N., Pan G., Zheng J., Wang R., Wang Y. (2019). Co_3_O_4_-ZnO p–n heterostructure nanomaterials film and its enhanced photoelectric response to visible lights at near room temperature. Mater. Res..

[cit50] Kahlaoui R., Sobrados I., Ternane R., Jimenez R., Sanz J. (2025). Li conductivity of Li_1+x_Ti_2_(PO_4_)_3-x_(SiO_4_)_x_ and Li_1_._2+x_Ti_1_._8_In_0_._2_(PO_4_)_3-x_(SiO_4_)_x_ (0≤x≤0.2) NASICON series. Composition and microstructure. Ceram. Int..

[cit51] Yu T., Chen Z., Wang Y., Xu J. (2021). Synthesis of ZnO-CuO and ZnO-Co_3_O_4_ materials with three-dimensionally ordered macroporous structure and its H_2_S removal performance at low-temperature. Processes.

[cit52] Liu H., Chen K., Feng Y. N., Chen F. F. (2023). In situ confined growth of Co_3_O_4_–TiO_2_/C S-scheme nanoparticle heterojunction for boosted photocatalytic CO_2_ reduction. J. Phys. Chem. C.

[cit53] Iqbal W., Mekki M., Rehman W. (2023). *et al.*, Electrical properties of TiO_2_/Co_3_O_4_ core/shell nanoparticles synthesised by sol-gel method. Dig. J. Nanomater. Bios.

[cit54] Mei D., Lu S., Liang L., Komarneni S., Ma J. (2026). Co_3_O_4_@CuO double core-shell hollow nanocages efficiently remove metronidazole by activating peroxymonosulfate under visible light. Colloids Surf., A.

[cit55] Rana A. K., Patel M., Nguyen T. T., Yun J.-H., Kim J. (2020). Transparent Co_3_O_4_/ZnO photovoltaic broadband photodetector. Mater. Sci. Semicond. Process..

[cit56] Reda G. M., Fan H., Tian H. (2017). Room-temperature solid-state synthesis of Co_3_O_4_/ZnO p–n heterostructure and its photocatalytic activity. Adv. Powder Technol..

[cit57] Ungeheuer K., Bocirnea A. E., Marszalek K. W. (2025). *et al.*, XPS study and electronic structure of nondoped and Cr^+^ ion implanted CuO thin films. Sci. Rep..

[cit58] Krishnan P., Liu M., Itty P. (2017). *et al.*, Characterisation of photocatalytic TiO_2_ powder under varied environments using near ambient pressure X-ray photoelectron spectroscopy. Sci. Rep..

[cit59] Alzaid M., Abu-Dief A. M., Hadia N., Ezzeldien M., Mohamed W. (2024). Synthesis and characterisation study of spinel Co_3_O_4_ nanoparticles synthesised via the facial coprecipitation route for optoelectronic application. Opt. Quantum Electron..

[cit60] Aftab U., Tahira A., Gradone A. (2021). *et al.*, Two step synthesis of TiO_2_–Co_3_O_4_ composite for efficient oxygen evolution reaction. Int. J. Hydrogen Energy.

[cit61] Liaqat M. A., Hussain Z., Khan Z. (2020). *et al.*, Effects of Ag doping on compact TiO_2_ thin films synthesised via one-step sol–gel route and deposited by spin coating technique. J. Mater. Sci.: Mater. Electron..

[cit62] Shahid M., Zhan Y., Sagadevan S. (2021). Cobalt oxide-based nanomaterial for electrochemical sensor applications. Malays. Nanoan Int. J..

[cit63] Reikowski F., Maroun F., Pacheco I. (2019). *et al.*, Operando surface X-ray diffraction studies of structurally defined Co_3_O_4_ and CoOOH thin films during oxygen evolution. ACS Catal..

[cit64] Alhashem Z. H. (2024). Ni-doped ZnO nanoparticles derived by the sol–gel method: Structural, optical, and magnetic characteristics. Arab. J. Chem..

[cit65] Sheng C. K., Aziz N. A. (2025). Ultraviolet-induced photoreduction of rhodamine 6G textile effluent catalysed by annealed metamorphic nano-CuO. Res. Chem..

[cit66] Chu Y., Feng J., Qian Y., Xiong S. (2015). Enhancing the electrode performance of Co_3_O_4_ through Co_3_O_4_@a-TiO_2_ core–shell microcubes with controllable pore size. RSC Adv..

[cit67] Dessie Y., Ravikumar R. C., Khasim S. (2025). *et al.*, Biosynthesised ZnO/Co_3_O_4_ nanocomposite-modified electrode for electrocatalytic lead detection and quantification. ACS Omega.

[cit68] Zhang W., Zeng T., Yu Y. (2025). *et al.*, Light-driven enhancement of oxygen evolution for clean energy conversion: Co_3_O_4_-TiO_2_/CNTs p–n heterojunction catalysts enabling efficient carrier separation and reduced overpotential. Energies.

[cit69] Ganga B. G., Santhosh P. N. (2014). Manipulating aggregation of CuO nanoparticles: Correlation between morphology and optical properties. J. Alloys Compd..

[cit70] Kirty A. S., Dutta S., Sharma R. K. (2026). *et al.*, Towards sustainable photocatalytic degradation of organic pollutants through rational design of engineered magnetically retrievable metal oxide@graphene oxide nanocomposites. Environ. Sci.: Adv..

[cit71] Bharali L., Mazumder M. H., Chakraborty D. (2024). *et al.*, Facile one-pot synthesis of a manganese–cerium dual-doped hydroxyapatite nanocatalyst using Phyllanthus emblica extract for photocatalytic degradation of methylene blue dye and catalytic reduction of 4-nitrophenol. New J. Chem..

[cit72] Al Miad A., Saikat S. P., Alam M. K. (2024). *et al.*, Metal oxide-based photocatalysts for the efficient degradation of organic pollutants for a sustainable environment: a review. Nanoscale Adv..

[cit73] Rabani I., Zafar R., Subalakshmi K. (2021). *et al.*, A facile mechanochemical preparation of Co_3_O_4_@g-C_3_N_4_ for application in supercapacitors and degradation of pollutants in water. J. Hazard. Mater..

[cit74] Dessie Y., Ravikumar R. C., Khasim S. (2025). *et al.*, Biosynthesised ZnO/Co_3_O_4_ nanocomposite-modified electrode for electrocatalytic lead detection and quantification. ACS Omega.

[cit75] Souamti A., Kahlaoui M., Mohammed B., Lozano-Gorrín A. D., Chehimi D. B. H. (2017). Synthesis, structural and electrochemical properties of new ytterbium-doped langbeinite ceramics. Ceram. Int..

[cit76] Souamti A., Kahlaoui M., Fezai R., Lozano-Gorrín A. D., Chehimi D. B. H. (2019). Synthesis, characterisation and electrical properties of Na_6_M(SO_4_)_4_ (M = Co, Ni, Cu) vanthoffite materials. Mater. Sci. Eng., B.

[cit77] Alharthi F., Hasan I. (2026). Cobalt oxide–copper oxide heterojunction nanocomposites for efficient UV-driven photocatalytic degradation of Brilliant Blue G dye. J. Sol-Gel Sci. Technol..

[cit78] Zhang L., Li H., Yang B. (2019). *et al.*, Photo-deposition of ZnO/Co_3_O_4_ core-shell nanorods with p–n junction for efficient oxygen evolution reaction. J. Solid State Electrochem..

[cit79] Souemti A., Nasri S., Sghaier R. B., Latrous L., Megriche A. (2024). Doping effects of transition metals and rare earth ions in KMPO_4_:Ln ceramics: Implications for electrical and microstructural characteristics. Mater. Chem. Phys..

[cit80] Lu W., Cai X., Jiao D. (2025). *et al.*, Electron transfer in heterojunctions comprised of Co_3_O_4_ nanorods decorated with CoP nanoparticles for electrocatalytic water splitting. ACS Appl. Nano Mater..

[cit81] Xu H., Zhu H.-R., Zhang Z.-J. (2024). *et al.*, Co/Co_3_O_4_ heterojunctions encased in porous N-doped carbon nanocapsules for high-performance cathode of rechargeable zinc-air batteries. Inorg. Chem..

[cit82] Liu S., Kang L., Hu J. (2021). *et al.*, Unlocking the potential of oxygen-deficient copper-doped Co_3_O_4_ nanocrystals confined in carbon as an advanced electrode for flexible solid-state supercapacitors. ACS Energy Lett..

[cit83] Souemti A., Labidi I., Megriche A. (2022). Spectroscopic properties and conduction mechanism of KAl(SO_4_)_2_:xSm: A multifunctional materials for optical and electrochemical applications. Ceram. Int..

[cit84] Gupta J., Ahmed A. S. (2022). Temperature-dependent analysis of dielectric behaviour of Co_3_O_4_/NiO nanocomposites with varying NiO concentration. J. Mater. Sci.: Mater. Electron..

[cit85] Szpakiewicz-Szatan A., Starzonek S., Garbarczyk J. (2024). *et al.*, AC electric conductivity of high pressure and high temperature formed NaFePO_4_ glassy nanocomposite. Nanomaterials.

[cit86] Moualhi K., Moualhi Y., Zouaoui M. (2024). Investigation of conduction mechanisms and permittivity–conductivity correlation in a Gd-based perovskite structure. RSC Adv..

[cit87] Moualhi Y., Nofal M. M., M'nassri R. (2020). *et al.*, Double Jonscher response and contribution of multiple mechanisms in electrical conductivity processes of Fe-PrCaMnO ceramic. Ceram. Int..

[cit88] Biswas D., Das A., Mondal R. (2020). *et al.*, Structural properties and electrical conductivity mechanisms of semiconducting quaternary nanocomposites: Effect of two transition metal oxides. J. Phys. Chem. Solids.

[cit89] Yassin A., Yassin A., Mohamed A., Abdel-Ghany A. M., Abdelrazek E. (2018). Enhancement of dielectric properties and AC electrical conductivity of nanocomposite using poly (vinyl chloride-co-vinyl acetate-co-2-hydroxypropyl acrylate) filled with graphene oxide. J. Mater. Sci.: Mater. Electron..

