# Supplementary material for: Versatile Co3O4@metal oxide nanocomposites: from photocatalytic pollutant degradation to high-efficiency energy supercapacitor storage
Source: RSC Adv. 2026 Sep 10;16(43):53838–58. doi: 10.1039/d6ra06628c (PMC13559541; doi:10.1039/d6ra06628c)
Supplement: Supplementary file 1 [file RA-016-D6RA06628C-s001.pdf]

This journal is © The Royal Society of Chemistry 2026

Supplementary materials

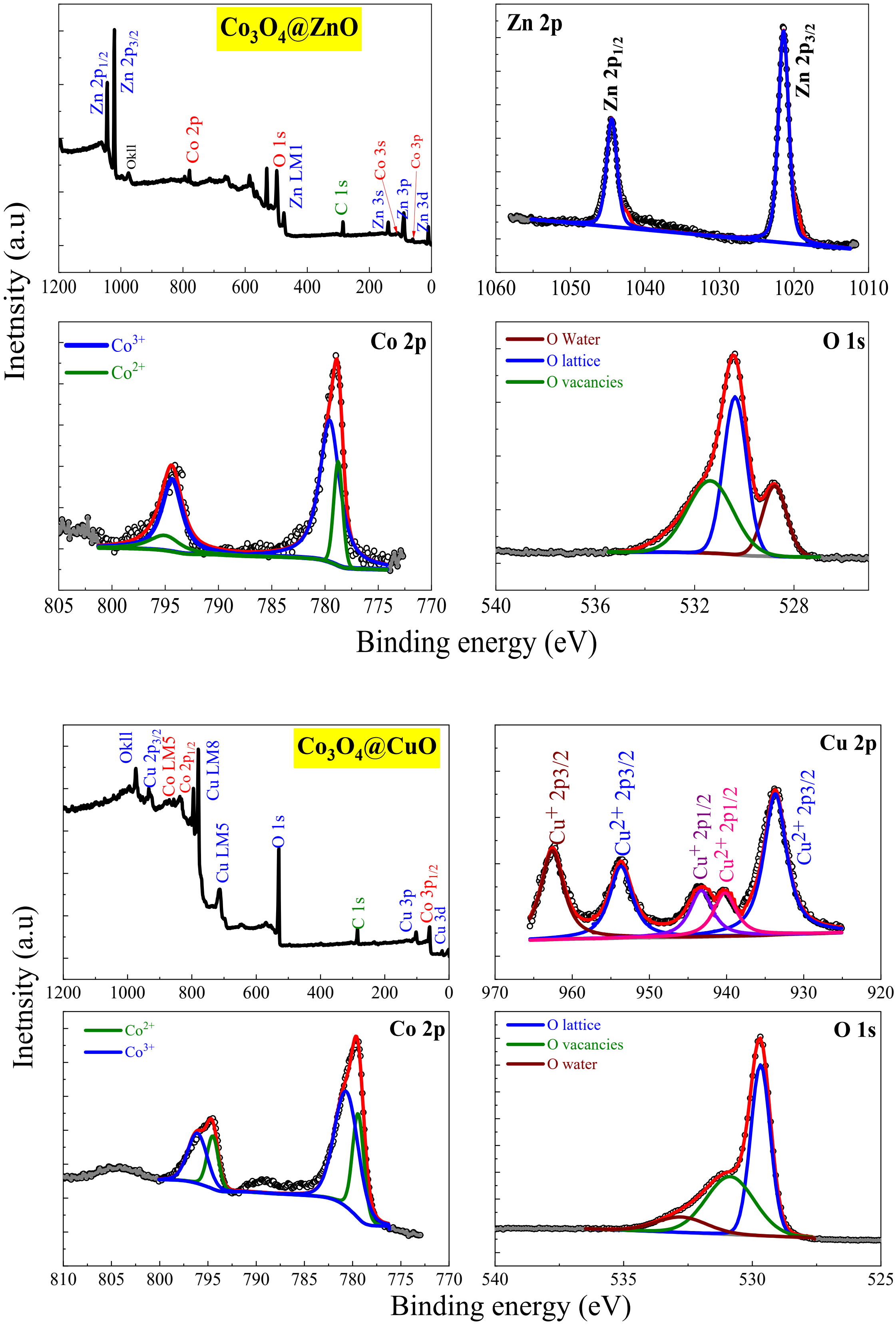

Fig. S1. XPS spectra of Co_3O_4@ZnO and Co_3O_4@TiO_2 nanocomposite materials.

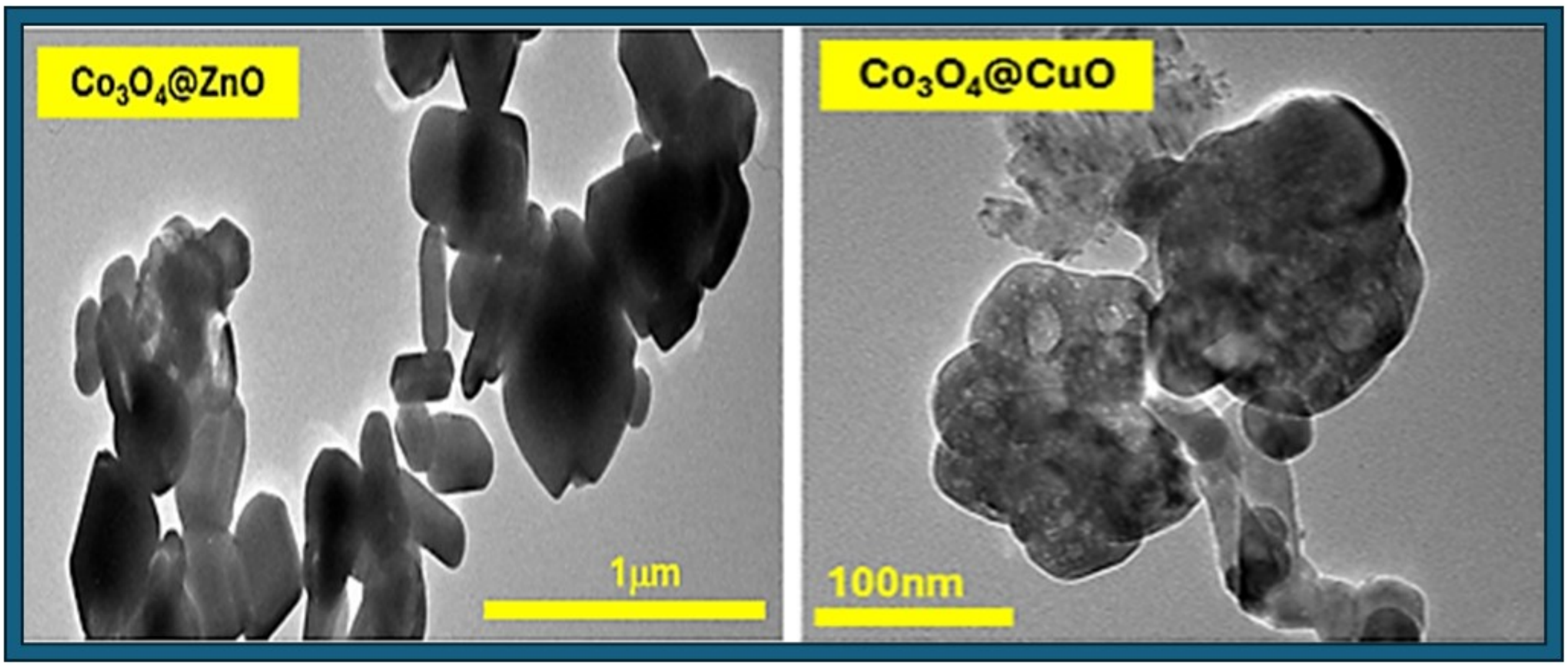

Fig. S2. TEM images of Co_3O_4@ZnO and Co_3O_4@CuO nanocomposite materials.

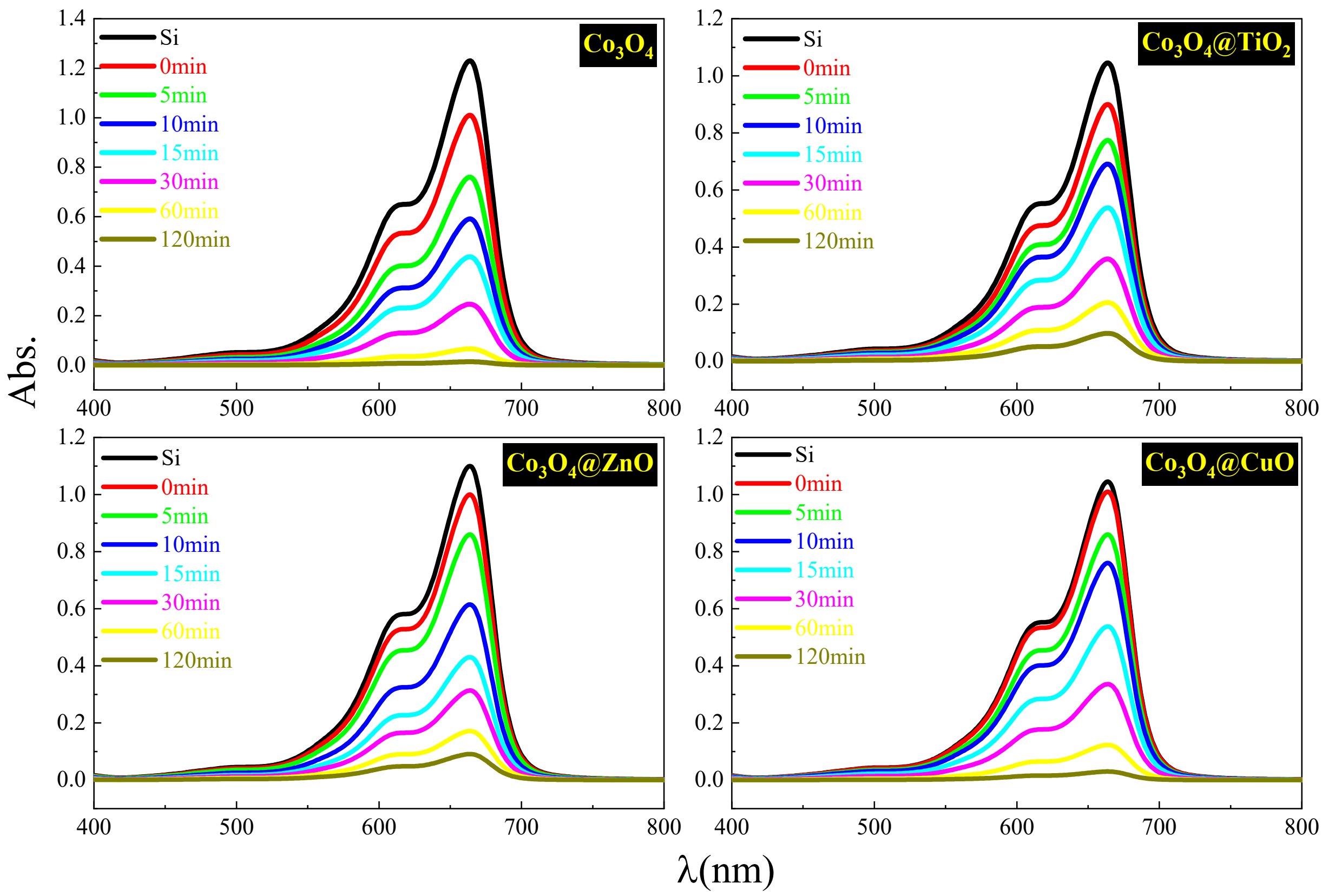

Fig. S3. Evolution of MB solution absorption as a function of sunlight time irradiation in the presence of Co_3O_4-based nanocomposite catalysts.

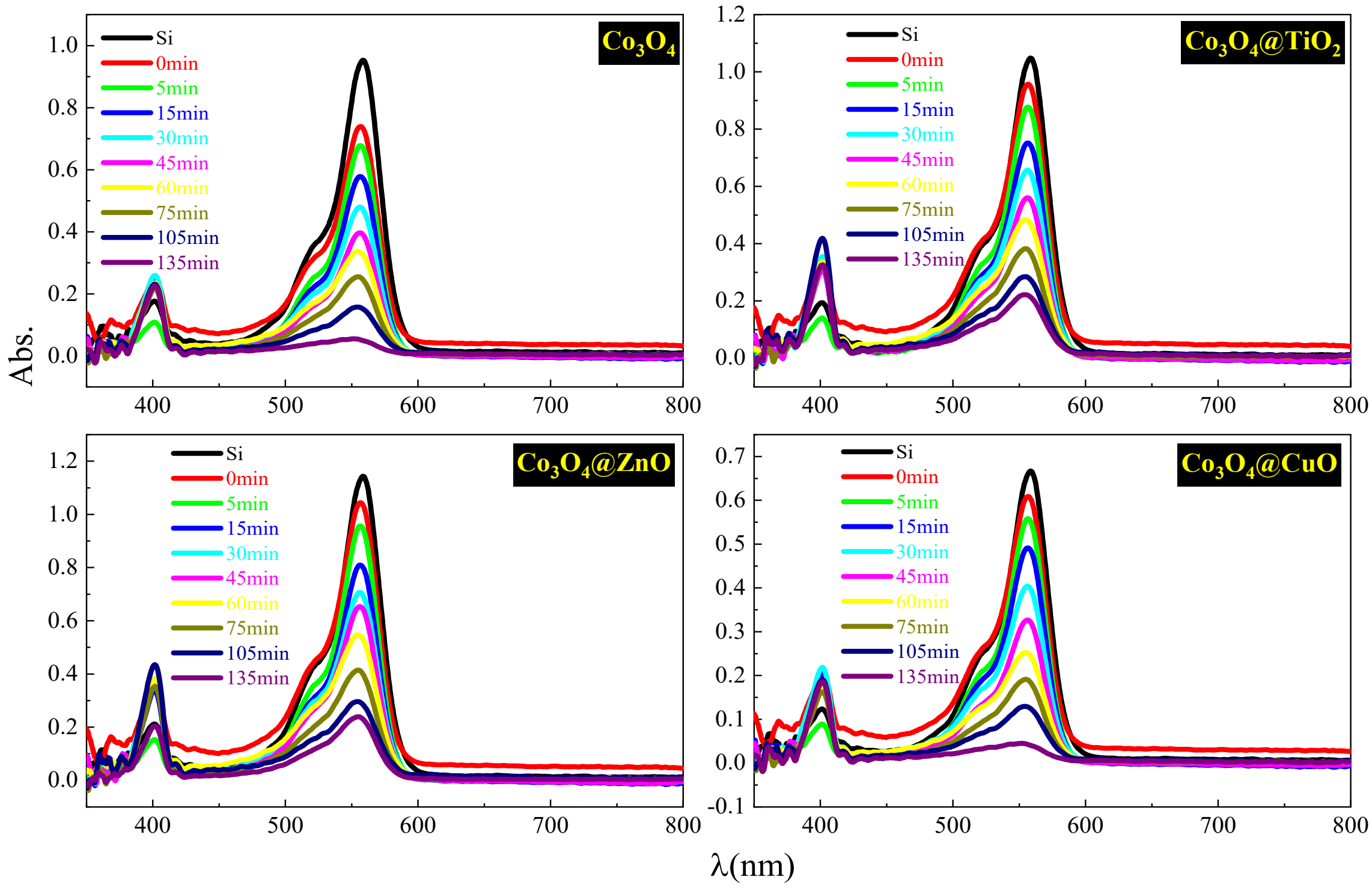

Fig. S4. Evolution of RB dye solution absorption as a function of solar irradiation time in the presence of Co_3O_4-based nanocomposite catalysts.
